# Efficacy and safety of Z-substances in the management of insomnia in older adults: a systematic review for the development of recommendations to reduce potentially inappropriate prescribing

**DOI:** 10.1186/s12877-022-02757-6

**Published:** 2022-02-01

**Authors:** Vincenz Scharner, Lukas Hasieber, Andreas Sönnichsen, Eva Mann

**Affiliations:** 1 Department of General Practice and Family Medicine, Center for Public Health, Medical University of Vienna, Vienna, Austria; 2 Institute of General Practice, Family Medicine and Preventive Medicine, Paracelsus Medical University, Salzburg, Austria

**Keywords:** Systematic review, Benzodiazepine-like medication, Z-drugs, Zolpidem, Insomnia, Inappropriate prescribing, Older people, Elder, Elderly

## Abstract

**Background:**

Z-drugs are usually prescribed as first line pharmacological therapy for insomnia. However, the benefits and risks of Z-drugs may differ for older adults. This systematic review investigated the available evidence on the efficacy and safety of Z-drugs in the management of insomnia in older adults.

**Methods:**

The Cochrane database of Systematic Reviews, the Cochrane Central Register of Controlled Trials, PubMed/MEDLINE and EMBASE were searched for systematic reviews, meta-analyses, controlled interventional and observational studies using a pre-formulated search term. The target population was older adults (≥65 years old) with insomnia. Studies were included if they reported efficacy and/or safety outcomes of the use of Z-drugs for the management of insomnia compared to placebo, usual or no treatment, or other pharmacological agents.

**Results:**

Eighteen studies were included (8 interventional and 10 observational studies). In short-term interventional studies, Z-drugs were similarly or better efficacious in improving both sleep and daytime parameters than placebo or other pharmacological treatments, while showing good results on measures of safety. However, in longer-term observational studies, Z-drugs significantly increased the risk for falls and fractures in comparison to no treatment or melatonin agonists.

**Conclusions:**

Analyzing the evidence from short-term interventional studies, Z-drugs appear effective and safe for treatment of insomnia in older adults, but they may have unfavorable side effects when used for longer periods of time. We, therefore, recommend discontinuing Z-drugs, principally because of the high risk for falls and fractures. Nonetheless, quality and quantity of evidence are low. Due to the scarcity of data, especially concerning drug dependence after longer periods of treatment and due to the significantly increased risk for falls and fractures, further studies are needed to evaluate the benefit-risk profile of Z-drugs use in older patients, particularly for long-term use.

**Supplementary Information:**

The online version contains supplementary material available at 10.1186/s12877-022-02757-6.

## Background

Insomnia is a major healthcare problem in the Western world. It is defined as a dissatisfaction with the quantity or quality of sleep and is associated with difficulties initiating or maintaining sleep as well as with early-morning waking with an inability to return to sleep [1]. Approximately 6 to 10% of adults experience insomnia that meets diagnostic criteria [2]. Insomnia is more commonly experienced by older adults and can occur independently or be caused by other diseases [3].

Benzodiazepine-like medications (BDLM), also called Z-drugs, are a chemically heterogenous group defined by their mechanism of action: a selectivity for certain γ- aminobutyric acid (GABA) receptor subunits that distinguishes them from Benzodiazepines (BDZ) [4–6].

Over the last 5 years zolpidem has been the most frequently prescribed hypnotic worldwide [7]. In the year of 2017, zolpidem and zopiclone were the top two prescribed BDLM in Europe [8]. Various studies confirm the high prescription rate of Z-drugs in community-dwelling, hospitalized and nursing home patients, with prescribing rates highest for older women [9–12].

BDLM are licensed only for short-term use. This restriction entails a paradox, as the vast majority of afflicted patients is in need of long-term treatment [13].

The time restriction on BDLM use is attributable to their effect-risk profile, where negative impact multiplies with prolonged duration of treatment and benefits decrease or stay steady at best [14].

BDLM were expected to achieve the strong sedative and hypnotic effects desired, while avoiding the anxiolytic, myorelaxant, analgesic, and anticonvulsant side effects of Benzodiazepines [7, 15, 16]. It was hypothesized that there was a link between receptor subtype selectivity and the reduction of side effects.

However, recent studies point to hang-over effects including impairment of cognitive and memory functions on the day after use [16, 17] the development of rebound insomnia after discontinuation of therapy, and most strikingly a lack of difference to BDZ in the rapid induction of tolerance [16], as well as a high risk of addiction resulting in an increasing proportion of chronic users and abusers [16, 18, 19].

In addition, observational studies linked Z-drugs to dementia and delirium, while demonstrating an association with car accidents and serious risks of falls and hip fractures [20].

Across North America, there have been several safety warnings for this class of medications, related to their use by older patients [21].

Controversy exists about the classification of Z-drugs as potentially inappropriate medication (PIM). The updated Beers Criteria by the American Geriatrics Society in 2019 strongly recommend strictly avoiding Z-drugs in older adults [22]. In the EU(7)-PIM list, Z-drugs are also classified as PIM, with the recommendation to choose the lowest dose (up to half of the usual dose) and the shortest possible duration of therapy. In contrast to these recommendations, Z-substances have not been classified as PIM in the Austrian PIM list, due to their inconsistent rating in previous literature; especially on the issue of dependency [23]. They are even featured as an alternative medication for BZD identified as PIM.

To the best of our knowledge, no systematic review (SR) has evaluated the evidence on the use of Z-drug to treat insomnia specifically in older adults.

The objectives of this SR are therefore to:

- review systematically the literature on the risks and benefits of the use of Z-substances in the treatment of insomnia in older adults
- critically assess the quality of evidence identified, and
- develop recommendations for or against the use of BDLM in the treatment of insomnia in older adults

## Methods

This systematic review was conducted in accordance with the methods developed in the Cochrane Handbook for Systematic Reviews of Interventions [24] and the Preferred Reporting Items for Systematic Reviews and Meta-Analyses (PRISMA) [25]. The study protocol was registered on PROSPERO and can be accessed under the registration number CRD42020156349.

### Study inclusion criteria

#### Types of studies

Systematic reviews, meta-analyses, controlled interventional studies and observational studies reporting on the safety and efficacy of the use of Benzodiazepine-like medication (BDLM) in the treatment of insomnia in older adults were included. We excluded abstracts, editorials, opinion papers, case reports, case series, narrative reviews, letters, qualitative studies and dose-response studies.

#### Types of participants

The population was defined as patients aged 65 or older with the indication for the prescription of BDLM. This age-threshold was chosen due to it being in standard use as it reflects retirement age in some developed countries [26, 27]. The inclusion criteria were:

For systematic reviews and meta-analyses:

- overall mean age − 1.2 SD ≥ 65 years; or
- overall mean age < 65 with subgroup analysis of participants with mean age − 1.2 SD ≥ 65 years; or
- overall mean age not reported but included studies accepting only participants ≥ 65 years

For individual controlled interventional and observational studies:

- mean age − 1.2 SD ≥ 65 years; or
- mean age < 65 with subgroup analysis of participants with mean age − 1.2 SD ≥ 65 years; or
- mean age not reported but all participants ≥ 65 years

#### Types of interventions

Studies reporting on the efficacy and/or safety of BDLM in all doses and formulations for the treatment of insomnia were included. Control was either no therapy, placebo, standard therapy, or other pharmacological or non-pharmacological interventions.

#### Types of outcomes

Outcomes included were quality of life, hospitalizations, mortality, falls, fractures, and severe organ failures.

Further outcomes that reflect the reduction of symptoms of insomnia and improvement in daytime function were included such as:

- Sleep latency
- Total sleep time
- Wake time after sleep onset
- Sleep quality
- Daily function
- Adverse events data

#### Setting

All settings were included.

#### Language

No language restrictions were included in the study searches.

### Search method

A comprehensive search for systematic reviews and meta-analyses, controlled interventional studies and observational studies was conducted using a predeveloped search term on four databases: PubMed/Medline, EMBASE, the Cochrane Database of Systematic Reviews and the Cochrane Central Register of Controlled Trials.

The PICOS-framework was used to develop a search term (population: people over 65 years, intervention: BDLM, comparator: no limits, outcomes: see list above ‘Types of outcomes’ and study design: systematic reviews, meta-analyses, controlled interventional studies and observational studies). The full search term can be found as Additional file 1.

The search was conducted on the 19th of June 2019 by a data research team at the University of Witten using the OVID interface for each database.

### Data management

Search results were saved on EndNote X9 reference management software. Upon retrieval, results were de-duplicated.

### Selection of studies

The titles and abstracts identified were independently screened for eligibility by two reviewers. Full text articles were obtained for all references meeting the inclusion criteria, or where there was uncertainty about inclusion. VS, LH and EM were involved in this task.

Consensus was established and in case of disagreement arbitrated by AS.

Reference lists of included studies and studies identified via snowballing were screened for eligibility.

Studies excluded in full text were listed with a justification for exclusion.

### Data extraction

Data extraction was conducted independently using a previously developed standardized data extraction form. Data items extracted were study design, objective, data of participants, intervention and comparator, study duration, outcome measures and sponsors. Completeness and accuracy of data extraction were double-checked by two further independent reviewers.

### Quality appraisal

For different study designs distinct validated tools of assessment were used to evaluate quality. Systematic reviews and meta-analyses were appraised using the critical appraisal tool for systematic reviews (AMSTAR2) [28], clinical studies utilizing the revised Cochrane risk-of-bias tool for randomized trials (RoB2) [29], and for observational studies the critical appraisal skills program checklist (CASP) [30] was used.

Quality appraisal was carried out by two independent researchers (VS and EM) and in case of disagreement arbitrated by a third reviewer (AS).

### Data synthesis

A descriptive and narrative summary of results with a focus on clinical endpoints was formulated. Quality of included studies was described. In case of data from included studies being homogenous enough in terms of treatments, study duration, study design and outcomes, a meta-analysis of results was calculated.

## Results

### Results of the search

Five hundred forty-two records were identified through database searches, and 33 additional records through other sources (hand searches of reference lists of included studies). After removing 26 duplicates, we screened 549 records and excluded 500 records scrutinizing titles and abstracts. We assessed 49 full text articles for eligibility and excluded 31 records. Main reasons for exclusion were wrong population age, wrong study design, and wrong publication type. A full list of excluded studies with reasons can be found as Additional file 2.

**Fig. 1:**
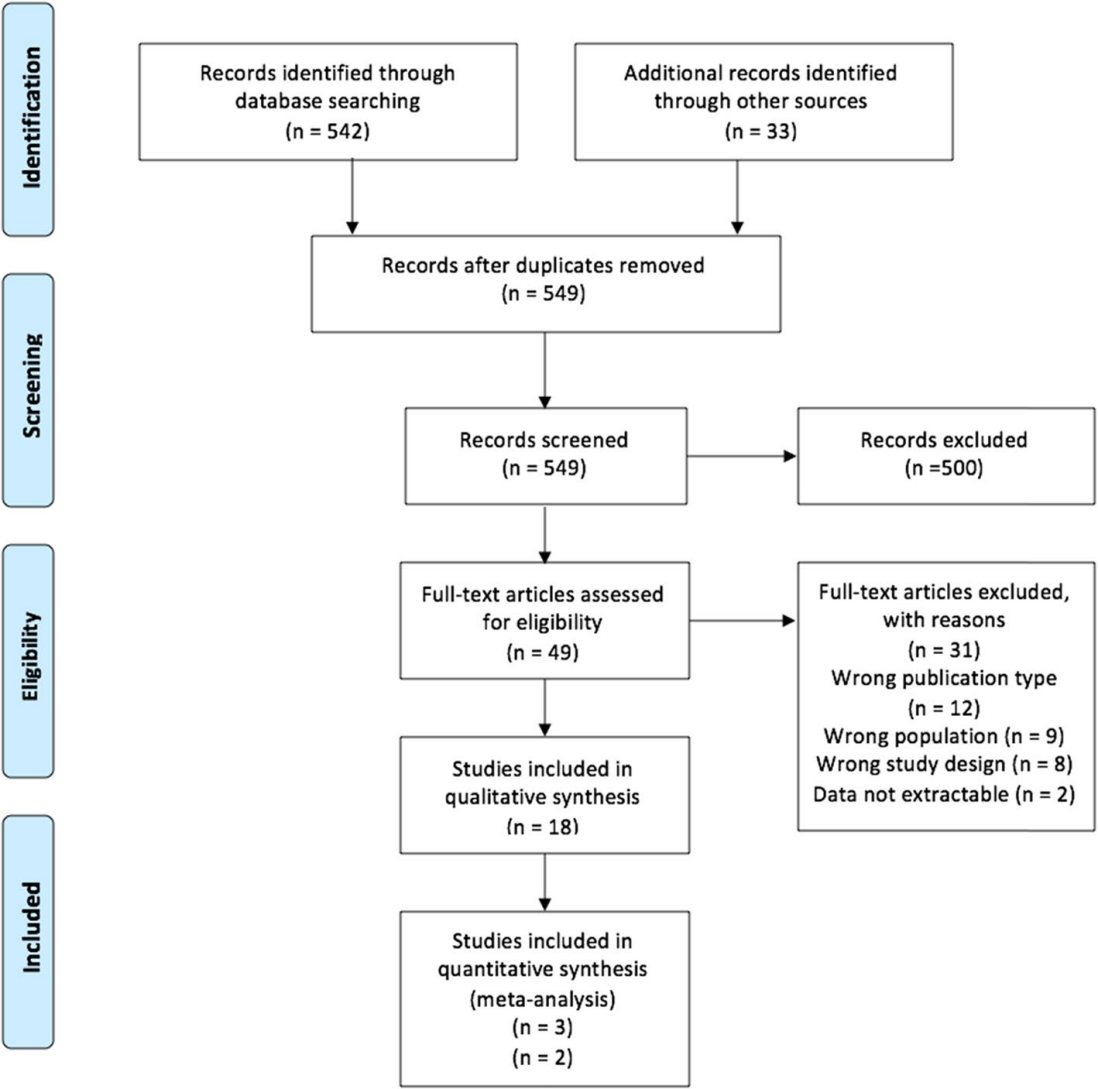
PRISMA flow diagram

The PRISMA flow diagram is presented in Fig. 1.

### Characteristics of included studies

The total of 18 eligible studies consists of eight randomized-controlled studies [31–38], nine case-control or case-crossover studies [39–47] and one cohort study [48]. Study characteristics are listed in Table 1.

**Table 1:** Summary of study characteristics

Authors and publication year	Study design	Aim	Sample size and number/percentage of patients > 65 years	Study duration or follow-up	Outcome	Sponsor
Ancoli-Israel et al. 2010 (66)	Randomized, placebo-controlled, double-blind study	To evaluate the efficacy and safety of 12 weeks of nightly eszopiclone in elderly outpatients with insomnia	*n* = 194 Eszopiclone 2 mg *n* = 194 placebo	2-week single-blind placebo assessment period 12-week treatment 2-week single-blind placebo run-out 2-week no drug follow-up	Sleep parameters Daytime function parameters Insomnia severity index (ISI) ADE	Sepracor INC., Marlborough, MA
Ancoli-Israel et al. 1999 (67)	Multicenter, randomized, placebo-controlled double-blind study	To evaluate the efficacy and safety of Zaleplon 5/10 mg in elderly outpatients with insomnia in comparison to Zolpidem 5 mg and placebo	*n* = 166 Zaleplon 5 mg *n* = 165 Zaleplon 10 mg *n* = 111 Zolpidem 5 mg *n* = 107 placebo	1-week single-blind placebo baseline period 2-week double-blind treatment 1-week single-blind placebo run-out	Sleep parameters Rebound insomnia ADE Routine physical examination (vital signs, ECG)	Wyeth-Ayerst Research, Radnor, Pa. and Zaleplon clinical study group
Avidan et al. 2010 (83)	Retrospective cohort analysis	To examine the risk of accidental events in older adults newly prescribed Benzodiazepine-like medication (BDLM) or selective melatonin receptor agonist (MRA)	*n* = 156,987 Benzodiazepine-like medication (Zolpidem, Zaleplon, Eszopiclone) *n* = 988 Selective melatonin receptor agonist (Ramelteon)	30 days of insomnia therapy and 3-month period after treatment initiation	Accidental events identified using External Causes of Injury Codes (ICD) and ICD-9-CM Codes in claims files	Takeda pharmaceutical company, Ltd
Berry et al. 2013 (74)	Case-crossover study	To estimate the association between BDLM use (Zolpidem, Eszopiclone, Zaleplon) and the risk for hip fractures among a nationwide sample of long-stay nursing home residents	*n* = 927 with discordant Exposure to BDLM in hazard and control periods	Dispensing of BDLM (Zolpidem, Eszopiclone, Zaleplon) 30 days before fracture (hazard period) or 60–90 days and 120–149 days before fracture (control periods)	Hip fracture defined as ICD-9: 820.xx or 733.14	National Institute on Aging and Friends of Hebrew Senior Life
Chang et al. 2011 (75)	Case-control study	To examine the association between medical conditions and medications and falls in older people in hospital	*n* = 19 Zolpidem *n* = 8 controls	Medications used within 24 h of falls	Falls reported in the hospital	Department of Health, Taiwan
Dehlin et al. 1995 (68)	Multicenter, randomized, double-blind study	To compare the efficacy and safety of Zopiclone 5 mg and Flunitrazepam 1 mg in elderly patients	*n* = 50 Zopiclone 5 mg *n* = 52 Flunitrazepam 1 mg	3-day single-blind placebo baseline period 2-week double-blind treatment 1-week single-blind placebo run-out	Sleep parameters Daytime function parameters ADE	Rhone-Poulenc Rorer, Box 33, Helsingborg Sweden
Elie et al. 1990 (69)	Randomized, placebo controlled, double-blind study	To evaluate and compare the efficacy and safety of Zopiclone 5/7.5 mg and Triazolam 0.125/0.25 mg in elderly patients with insomnia in comparison with placebo	*n* = 15 Zopiclone 5 mg for 1 week then increase to 7.5 mg for 2 weeks *n* = 14 Triazolam 0.125 mg for 1 week then increase to 0.25 mg for 2 weeks *n* = 15 placebo	3-day single-blind wash-out period 3-week double-blind treatment period 4-day single-blind wash-out period	Sleep parameters Hang-over Routine physical examination (ECG, routine lab test) ADE	Rhone-Poulenc Pharma Inc., Montreal, CA
Kang et al. 2012 (76)	Case-crossover study	To evaluate the risk of fractures related with Zolpidem in elderly insomnia patients	*n* = 1508 with discordant exposure to BDLM in hazard and control periods	Prescription of Zolpidem 1 day before fracture (hazard period) or 1 day before 5/10/15 and 20 weeks before fracture (control periods)	Diagnosis of fracture ICD-10: S02, S12, S22, S32, S42, S52, S62, S72, S82, S92, T02	No information
Klimm et al. 1987 (70)	Randomized double-blind study	To evaluate the efficacy and safety of Zopiclone 7.5 mg in elderly patients with insomnia in comparison to Nitrazepam 5 mg	*n* = 36 Zopiclone 7.5 mg *n* = 36 Nitrazepam 5 mg	1-week single-blind placebo wash-out period 1-week double-blind treatment	Sleep parameters Spiegel Sleep Questionnaire ADE General evaluation	No information
Lai et al. 2015 (77)	Case-control study	To investigate the correlation between zopiclone use and risk of hip fracture in elderly adults	Cases: *n* = 170 Zopiclone last 7 days *n* = 780 Zopiclone before *n* = 10,046 non-users Controls: *n* = 37 Zopiclone last 7 days *n* = 586 late users *n* = 8458 non-users	Prescription of Zopiclone: − 7 days prior to fall -any time before -never	First episode of hip fracture according to ICD 9	Taiwan Ministry of Health and Welfare Clinical Trial Research Center of Excellence, China Medical University Hospital Academia Sinica of the Taiwan Biobank Stroke Biosignature Project of the National Research Program for Biopharmaceuticals Stroke Clinical Trial Consortium, Taiwan Tseng-Lien Lin Foundation, Taiwan Tai- wan Brain Disease Foundation, Taiwan Katsuzo and Kiyo Aoshima Memorial Funds, Japan
Leppik et al. 1997 (71)	Randomized, placebo-controlled double-blind study	To evaluate the efficacy and safety of Zolpidem 5 mg, Triazolam 0.125 mg and Temazepam 15 mg in elderly patients with insomnia in comparison with placebo	*n* = 76 Zolpidem 5 mg *n* = 71 Triazolam 0.125 mg *n* = 74 Temazepam 15 mg *n* = 74 placebo	1-week single-blind placebo wash-out period 4-week double-blind treatment	Sleep parameters Daytime function parameters Global Impression of Therapy score ADE Routine physical examination (ECG, laboratory evaluation)	Lorex Pharmaceuticals, Skokie, IL
Pierfitte et al. 2001 (78)	Case-control study	To determine whether benzodiazepines are associated with an increased risk of hip fracture	Cases: Zolpidem *n* = 15 Zopiclone *n* = 14 Controls: Zolpidem *n* = 35 Zopiclone *n* = 48	Drug use 24 h before fracture determined from questionnaires, medical records, or blood samples	Acute hip fracture resulting from a fall, not associated with cancer, a traffic accident, or aggression, ascertained from x-ray films and orthopedic consultation	Agence du médicament, Paris Department of Pharmacology, Victor Segalen University, Bordeaux, France
Roger et al. 1993 (72)	Randomized placebo- controlled double-blind multicenter study	To compare the efficacy and safety of Zolpidem with a reference benzodiazepine in elderly insomniac patients	*n* = 70 Zolpidem 5 mg *n* = 74 Zolpidem 10 mg *n* = 77 Triazolam 0.25 mg	3-day placebo wash-out period 3-week double-blind treatment period 7-day placebo withdrawal period	Sleep parameters Use of rescue hypnotic medication ADE	No information
Scharf et al. 2005 (73)	Randomized placebo- controlled double-blind multicenter study	To evaluate the efficacy of Eszopiclone and primary insomnia	*n* = 72 Eszopiclone 1 mg *n* = 79 Eszopiclone 2 mg *n* = 80 placebo	2-week double-blind treatment period	Sleep parameters Daytime function parameters Q-LES-Q ADE Routine physical examination (ECG, vital signs, neurological examination, lab measures)	Sepracor, Inc., Marlborough, Mass., US
Tang et al. 2015 (79)	Case-crossover study	To clarify the risk of acute effects of Zolpidem on all-sites of fracture in the elderly	*n* = 6010 with discordant exposure to BDLM in hazard and control periods	1 day prior to fracture (hazard period) or 1 day 5/10/15/20 weeks prior to fracture (control periods)	ICD-9 CM: 820, 812, 813, 814, 805, 806, 800–829	Tri-Service-General-Hospital Ministry of Science and Technology of Taiwan National Chiao Tung University Ministry of Education of Taiwan
Tom et al. 2016 (80)	Case-crossover study	To test the hypothesis that use of Zolpidem, Eszopiclone, and Zaleplon would be associated with increased risk of traumatic brain injury (TBI) and hip fracture	TBI: BDLM users *n* = 491 non-BDLM users *n* = 14,540 Hip fracture: BDLM users *n* = 1192 non-BDLM users *n* = 36,641	30 days before fracture (hazard period) or 3/6/9/12 months before fracture (control periods)	ICD-9 CM: 800.xx, 801.xx, 803.xx, 804.xx, 850.xx-854.1x, 950.1–950.3, 959.01; 820.xx	National Institute of Health
Wang et al. 2001 (81)	Case-control study	To identify hazardous regimens (for insomnia) that should be avoided and safer regimens that may be used preferentially by older people	*n* = 20 Zolpidem *n* = 35 controls	Index date and 6 months prior	Hospitalization for surgical repair of hip fracture	National Institute of Drug Abuse National Institute on Aging
Zint et al. 2010 (82)	Case-control study	To determine how concomitant use of potentially interacting drugs, drug dosage, and duration of therapy modify the risk of hip fracture associated with use of BDLM in older adults	Cases: Zaleplon *n* = 21 Zolpidem *n* = 456 Controls: Zaleplon *n* = 62 Zolpidem *n* = 1608	14 days before fracture	First hip fracture leading to hospitalization	Graduiertenkolleg 793 National Institute on Aging of the National Institutes of Health Federal Ministry of Education and Research

### Patient characteristics

#### RCTs

In summary, 1902 persons participated in the RCTs. All studies analyzed participants older than 65 years [31–38]. The proportion of male participants ranged from 19.2% [35] to 45.6% [38]. Four trials reported ethnicities [31, 32, 36, 38].

The number of participants ranged from 44 [34] to 549 [32]. Study duration varied from 2 weeks [35, 38] to 18 weeks [31].

Four trials reported comorbid conditions [31, 33, 35, 38], co-medication was mentioned in 5 studies [31, 33–35, 38], and cognitive examination was performed in 5 trials [31–33, 35, 38]. All RCTs included aimed at evaluating the efficacy and safety of BDLM either versus placebo [31, 32, 38] or versus BDZ [33, 35, 37], or versus placebo and BDZ [34, 36].

Four RCTs were conducted in the US [31, 32, 36, 38], one was conducted in Sweden [33], one in Canada [34], one in Germany [35], and one study was carried out in France and Belgium [37]. Six studies were sponsored by pharmaceutical companies [31–34, 36, 38], in the remaining two studies [35, 37] no information was provided about sponsoring.

#### Observational studies

One retrospective cohort study was included in our analysis [48]. All participants were older than 70 years and up to 60% were female. The study included 156,987 participants taking BDLM and aimed at examining the risk of accidental events. Data were recorded over a period of 3 months following a period of at least 3 months without a prescription claim for insomnia medication. The study provided information about comorbidities and co-medication, but no data about physical or cognitive examinations is given. It was conducted in the US and was carried out from 2000 to 2006.

Five case-control [40, 42, 43, 46, 47] and four case-crossover studies [39, 41, 44, 45] were included in our analysis. In total, 83,727 participants took part in the nine studies. The number of participants varied widely ranging from 27 [40] to 20,077 participants [42]. All participants were older than 65 years.

Three studies did not report on the sex of participants [40, 43, 47], in the remaining six trials [39, 41, 42, 44–46] the proportion of male participants ranged from 16% [46] to 40% [42].

Kang, Pierfitte, Tang and Zint provided no information about ethnicity. Six studies [39, 41, 42, 44–46] delivered information about comorbidities, while four studies [41, 42, 45, 46] gave notice about co-mediation. One study [43] reported findings of physical and cognitive examinations.

Outcome parameters were fractures [41, 44], hip fractures [39, 42, 43, 45–47], falls in the hospital [40], and traumatic brain injury [45].

Four studies were conducted in the US [39, 45–47], three in Taiwan [40, 42, 44], one in South Korea [41], and one in France [43].

Additional file 3 shows the characteristics of the participants of the included studies.

### Quality appraisal and study quality

#### RCTs

The year of publication of the included RCTs ranges from 1987 to 2010. A summary of the risk of bias assessments for the RCTs is displayed in Table 2.

**Table 2:** Risk of bias assessment of randomized controlled trials

Study	Randomization process	Deviations from intended interventions	Missing outcome data	Measurement of the outcome	Selection of reported results	Overall risk
Ancoli-Israel 2010	Unknown risk	Some concerns	Low risk	Low risk	Low risk	High risk
Ancoli-Israel 1999	Unknown risk	High risk	Low risk	Some concerns	Unknown risk	High risk
Dehlin 1995	Unknown risk	High risk	Low risk	Unknown risk	Unknown risk	High risk
Elie 1990	Unknown risk	High risk	Some concerns	Unknown risk	Unknown risk	High risk
Klimm 1987	Unknown risk	Unknown risk	Low risk	Unknown risk	Unknown risk	High risk
Leppik 1997	Unknown risk	High risk	Low risk	Unknown risk	Unknown risk	High risk
Roger 1993	Unknow risk	High risk	Some concerns	Unknow risk	Unknown risk	High risk
Scharf 2005	Unknown risk	High risk	Low risk	Unknown risk	Low risk	High risk

The overall risk of all included randomized-controlled studies was classified as high [31–38].

The randomization process remained unclear in all included RCTs. Allocation concealment was clearly inadequate in the study of Ancoli-Israel 2010 and turned out to be unclear in the remaining 7 RCTs [31–38]. The studies of Ancoli-Israel 1999, Dehlin, Elie, Leppik, Roger and Scharf showed a high risk for deviations from intended interventions. Two studies [34, 37] maintained some concerns in respect to missing outcome data, while bias from missing outcome data was interpreted as being low in all other six studies [31–33, 35, 36, 38].

Concerning the measurement of the outcome only the study of Ancoli-Israel 2010 revealed a low risk; it remained unknown in studies published by Dehlin, Elie, Klimm, Leppik, Roger and Scharf.

Finally, the two studies published by Ancoli-Israel 2010 und Scharf showed low risk in terms of selection of reported outcome, whereas the risk was assessed as unknown in the remaining six studies [32–37].

#### Observational studies

The publication years of the observational studies range from 2001 to 2016.

A summary of the risk of bias assessment checklists for the nine case-control and case-crossover studies is depicted in Table 3 and for the single included cohort study in Table 4.

**Table 3:** Risk of bias of case-control and case-crossover studies

Study	Clear focus of research	Appropriate method of measurement	Acceptable recruitment of cases	Acceptable selection of controls	Accurate measurement of exposure	Equal treatment of groups	Identification and consideration of confounding factors	Size of treatment effect	Precision of estimate	Believability of results	Local application	Confirms available research
Berry 2013	Yes	Yes	Yes	Unknown	Yes	Yes	Yes	OR 1.47	CI95* 1.24–1.74	Yes	Unknown	Yes
Chang 2011	Yes	Yes	Yes	No	Yes	Yes	No	OR 2.38	CI95 1.04–5.43	No	Unknown	Yes
Kang 2012	Yes	Yes	Yes	Unknown	Yes	Yes	Yes	OR 1.84	CI95 1.47–2.3	Yes	Unknown	Partially
Lai 2015	Yes	Yes	Yes	No	Yes	Yes	No	OR 3.87	CI95 2.71–5.53	No	Unknown	Yes
Pierfitte 2001	Yes	Yes	Yes	No	Yes	Yes	No	OR 1.3	CI95 0.7–2.5	Yes	Yes	Yes
Tang 2015	Yes	Yes	Yes	Unknown	Yes	Yes	Yes	OR 1.23	CI95 1.06–1.44	Yes	Unknown	Yes
Tom 2016	Yes	Yes	Yes	Unknown	Yes	Yes	Yes	OR 1.87	CI95 1.56–2.25	Yes	Unknown	Yes
Wang 2001	Yes	Yes	Yes	No	Yes	Yes	No	OR 2.26	CI95 1.28–3.97	No	Unknown	Yes
Zint 2010	Yes	Yes	Yes	No	Yes	Yes	No	RR 1.48	CI95 1.32–1.66	No	Yes	Yes

* CI95 95% Confidence interval

**Table 4:** Risk of bias of the cohort study

Study	Clear focus of research	Acceptable recruitment of cohort	Accurate measurement of exposure	Accurate measurement of outcome	Identification and consideration of confounding factors	Appropriate length and scale of follow-up	Results and precision	Believability of results	Local application	Confirms available research
Avidan 2010	Yes	Yes	Yes	Yes	Yes	Unknown	OR 1.12 after 1 month OR 1.48 after 3 months (CI95 n.r.) *P* < 0.05	Yes	Yes	Yes

Most of the included studies reported sufficient detail to assess their quality. All studies [39–47] showed a clear focus of research, an appropriate method of measurement, an acceptable recruitment of cases, an accurate measurement of exposure, and equal treatment of groups. All studies but one [41] confirmed the available evidence. Believability of the results was provided by 5 of the included studies [39, 41, 43–45]. Major confounding factors were identified in 4 studies [39, 41, 44, 45]. All studies reported on the size of treatment effects and precision of estimate.

The single included cohort study [48], retrospective in nature, provided no information on the appropriate length and scale of follow up. Quality in all other items included in the quality appraisal was good.

### Efficacy and benefit

All RCTs assessed data about the efficacy and/or effectiveness of BDLM using subjective reports of sleep parameters and quality.

Outcome parameters included in the RCTs were sleep latency, total sleep time, wake time after sleep onset, numbers of awakenings, sleep quality, and daytime parameters.

Five studies found a significant decrease in SL as compared to placebo [31, 34–36, 38] and an advantage in comparison to triazolam and temazepam [34, 36]. A significant increase of total sleep time was reported in the studies published by Leppik, Roger and Scharf. Whilst the number of awakenings under treatment with BDLM was significantly lower when compared to placebo [38], there was no difference in comparison with flunitrazepam [33] and triazolam [37].

### Safety and adverse effects

The multiple adverse events of treatment with BDLM in the RCTs included dizziness [31, 35–37], nervousness [31, 36], falls [31, 37], anxiety, memory impairment and hallucinations [31], confusion [35], and fatigue [36].

All five case-control [40, 42, 43, 46, 47] and four case-crossover studies [39, 41, 44, 45] as well as the retrospective cohort study [48] focused on predefined health problems and their association to BDLM treatment.

Six studies provided data on an association between BDLM treatment and hip fractures [39, 42, 43, 45–47], two studies reported on fractures overall [41, 44], one study focused on traumatic brain injury [45], another one researched the connection between BDLM treatment and falls [40] and a third examined the relationship with all types of accidental events [48].

In these studies, an increased risk for hip fractures (OR range 1.3 (CI95 0.7–2.5); 3.87 (CI95 2.71–5.53)), traumatic brain injury (OR 1.87 (CI95 1.56–2.25)), fractures (OR range 1.84 (CI95 1.47–2.30); 1.27 (CI95 1.09–1.48)), falls and fractures (OR 2.38 (CI95 1.04–5.43)) and accidental events (OR of 1.12 (CI95 n.r.)) was reported.

A summary of the findings of controlled and observational studies can be found as Additional files 4 and 5, respectively.

### Meta-analyses

**Fig. 2:**
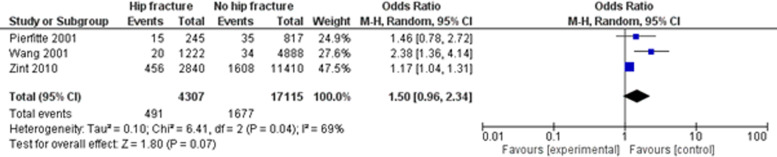
Zolpidem use and hip fracture risk

Due to heterogeneity of the included studies only two meta-analyses could be performed. The first meta-analysis includes three case-control-studies [43, 46, 47] investigating Zolpidem use in patients with and without hip fracture (see Fig. 2). In this meta-analysis, significance is just missed.

**Fig. 3:**
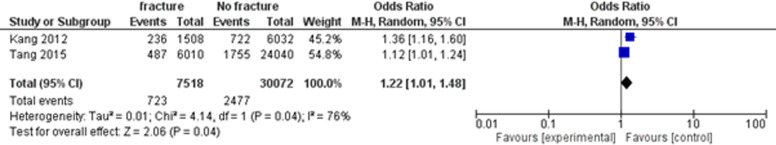
Any fracture and Zolpidem use

The second meta-analysis studies the risk of any fracture in users and non-users of Zolpidem in two studies [41, 44] with a case-crossover-design (see Fig. 3). The meta-analysis reveals a significant relationship between fracture risk and zolpidem use.

Both meta-analyses are characterized by a high heterogeneity of the included studies (I^2^ > 50%).

### Recommendation

A GRADE Evidence Profile table and a GRADE Summary of Findings table were created to summarize the results of this systematic review and are shown here as Tables 5 and 6, respectively.

**Table 5:** GRADE evidence profile

Certainty assessment							№ of patients		Effect		Certainty	Importance
№ of studies	Study design	Risk of bias	Inconsistency	Indirectness	Imprecision	Other considerations	BDLM	Placebo	Relative (95% CI)	Absolute (95% CI)		
Mortality												
0									not estimable		–	CRITICAL
Hospitalization (timing of exposure: 30 days; assessed with: number of cases with hospitalization for traumatic brain injury and hip fractures)												
3 (80–82)	observational studies										–	CRITICAL
Quality of life (Eszopiclone vs. placebo) (follow up: mean 12 weeks; assessed with: SF-36)												
1 (66)	randomized trials	serious ^a^	not serious	not serious	not serious	none	194	194	–	mean **71.6 higher** (0 to 0)	⨁⨁⨁◯ MODERATE	CRITICAL
Accidental event (follow up: mean 3 months; assessed with: events)												
1 (83)	observational studies	not serious	not serious	not serious	not serious	none	440/156,987 (0.3%)	0.5/988 (0.1%)	**OR 2.77** (CI95 0.38 to 19.76)	**1 more per 1000** (from 0 fewer to 9 more)	⨁⨁⨁⨁ HIGH	CRITICAL
Hip fracture with Zolpidem (timing of exposure: range 1 day to 180 days; assessed with: events)												
3 (78, 81, 82)	observational studies	not serious	not serious	not serious	not serious	none	4307 cases 17,115 controls		**OR 1.50** (CI95 0.96 to 2.34)	–	⨁⨁⨁⨁ HIGH	CRITICAL
Any fracture with Zolpidem (timing of exposure: mean 1 day; assessed with: events)												
2 (76, 79)	observational studies	not serious	not serious	not serious	not serious	none	7518 cases 30,072 controls		**OR 1.22** (CI95 1.01 to 1.48)	–	⨁⨁⨁⨁ HIGH	CRITICAL
Falls (timing of exposure: mean 1 day; assessed with: events)												
1 (75)	observational studies	serious ^b^	not serious	not serious	not serious	none	165 cases 165 controls		**OR 2.38** (CI95 1.04 to 5.43)	–	⨁⨁⨁◯ MODERATE	CRITICAL
Effect “well-rested” (follow up: 12 weeks; assessed with: sleep quality 11-point Likert scale Eszopiclone 2 mg vs placebo; Scale from: 0 to 11)												
1 (73)	randomized trials						79	80	–	SMD **7.1 SD higher** (0 to 0)	–	CRITICAL

**Table 6:** GRADE summary of findings

Outcomes	Anticipated absolute effects^*^ (95% CI)		Relative effect (95% CI)	№ of participants (studies)	Certainty of the evidence (GRADE)	Comments
	**Risk with placebo**	**Risk with BDLM**				
Mortality	0 per 1000	**0 per 1000** (0 to 0)	not estimable	(0 studies)	–	Mortality was not assessed in any of the studies
Hospitalization assessed with: number of cases with hospitalization for traumatic brain injury and hip fractures timing of exposure: 30 days				20,103 cases 142,059 controls (3 observational studies) (80–82)	–	Tom 2016 showed an OR of 1.87 (CI95 1.56–2.25) for hospitalization for traumatic brain injury in Zolpidem users vs. non-users. ORs were 0.67 (CI95 0.40–1.13) for Eszopiclone and 0.85 (CI95 0.21–3.34) for Zaleplon. Hospitalization for hip fracture was investigated by Tom 2016, Wang 2001 and Zint 2010. ORs for Zolpidem were 1.59 (CI95 1.41–1.79), 1.95 (CI95 1.09–3.51) and 1.26 (CI95 1.11–1.44), for Eszopiclone 1.12 (CI95 0.83–1.50), and for Zaleplon 0.92 (CI95 0.40–2.13).
Quality of life (Eszopiclone vs. placebo) assessed with: SF-36 follow up: mean 12 weeks	The mean quality of life (Eszopiclone vs. placebo) was **68.5**	mean **71.6 higher** (0 to 0)	–	388 (1 RCT) (66)	⨁⨁⨁◯ MODERATE ^a^	Score for General health provided (*p* = 0.009). Score for vitality also better for Eszopiclone [58.9 (21.2 vs. 55.1 (20.3), *p* = 0.008], all other SF-36-scores (domains) no significant differences.
Accidental event assessed with: events follow up: mean 3 months	1 per 1000	**1 per 1000** (0 to 10)	**OR 2.77** (0.38 to 19.76)	157,975 (1 observational study) (83)	⨁⨁⨁⨁ HIGH	The adjusted OR was 1.48, *p* < 0.05, 95% CI not provided.
Hip fracture with Zolpidem assessed with: events timing of exposure: range 1 days to 180 days	98 per 1000	**140 per 1000** (94 to 203)	**OR 1.50** (0.96 to 2.34)	4307 cases 17,115 controls (3 observational studies) (78, 81,82)	⨁⨁⨁⨁ HIGH	There were three more studies evaluating fracture risk which could not be included in the meta-analysis. Tom 2016 showed an increased hip fracture risk for Zolpidem, OR 1.59 (CI95 1.41–1.79), but not for Eszopiclone and Zaleplon. Lai 2015 revealed an increased hip fracture risk for Zopiclone, aOR 3.56 (CI95 2.33–4.84), Berry 2013 showed an increases hip fracture risk for all Z-substance users, OR 1.66 (CI95 1.45–1.90). (74, 77, 80)
Any fracture with Zolpidem assessed with: events timing of exposure: mean 1 days	82 per 1000	**99 per 1000** (83 to 117)	**OR 1.22** (1.01 to 1.48)	7518 cases 30,072 controls (2 observational studies) (76, 79)	⨁⨁⨁⨁ HIGH	Tang 2015 provides an adjusted OR of 1.13 (CI95 0.96–1.34) and Kang 2012 an adjusted OR of 1.72 (CI95 1.37–2.16).
Falls assessed with: events timing of exposure: mean 1 days	48 per 1000	**108 per 1000** (50 to 217)	**OR 2.38** (1.04 to 5.43)	165 cases 165 controls (1 observational study) (75)	⨁⨁⨁◯ MODERATE^b^	Results are based on only one case-control-study with high risk of bias.
Effect “well-rested” assessed with: sleep quality 11-point Likert scale Eszopiclone 2 mg vs Placebo Scale from: 0 to 11 follow up: 12 weeks	–	SMD **7.1 SD higher** (0 to 0)	–	159 (1 RCT) (73)	–	

*CI* Confidence interval, *OR* Odds ratio, *SMD* Standardized mean difference

^*^The risk in the intervention group (and its 95% confidence interval) is based on the assumed risk in the comparison group and the relative effect of the intervention (and its 95% CI)

Explanations: ^a^ randomization process, concealment of allocation, and blinding unclear; ^b^ High risk for selection bias, no adjustment for confounders

Explanations: a. randomization process, concealment of allocation, and blinding unclear; b. High risk for selection bias, no adjustment for confounders.

*CI* Confidence interval, *OR* Odds ratio, *SMD* Standardized mean difference.

Based on the results of the included studies and additional references of interest, one recommendation in relation to BDLM use in older adults with insomnia was developed. The recommendation is that clinicians should consider discontinuing longer term use of BDLM, principally because of the high risk for falls and fractures. The recommendation was considered a strong recommendation. The quality was downgraded from high to low because the evidence was derived from case-control and other observational studies only [39–49].

## Discussion

To the best of our knowledge, our systematic review is the first to evaluate the evidence on the use of Z-drugs to treat insomnia specifically in older adults. We included eight RCTs [31–38], nine case-control and case-crossover studies [39–47], and one retrospective cohort study [48].

Five RCTs found a significant decrease in SL as compared to placebo [31, 34–36, 38] and an advantage in comparison to triazolam and temazepam [34, 36]. A significant increase of total sleep time was reported in the studies published by Leppik, Roger and Scharf. Whilst the number of awakenings under treatment with BDLM was significantly lower when compared to placebo [38], there was no difference in comparison with flunitrazepam [33] and triazolam [37].

However, important limitations concerning the evidence on efficacy and safety of BDLM in the included RCTs must be taken into consideration. Study duration was short, varying from only 2 weeks to 18 weeks, no RCT addressed the problems of dependency and induction of tolerance, a major medication issue as most older patients who suffer from insomnia are chronic users of BDLM, and only four RCTs reported comorbid conditions [31, 33, 35, 38], while five RCTs mentioned co-medication [31, 33–35, 38] and an examination of cognitive status [31–33, 35, 38].

The overall study quality of the RCTs must be considered low, particularly in terms of the randomization process and risk of selection of reported outcomes. In addition, six out of the eight RCTs were sponsored by pharmaceutical companies and for the further two no information about sponsors was provided.

A study conducted by Ancoli-Israel et al., which could not be included in our review due to the lack of comparator, investigated zopiclone treatment for the longer period of 6 to 12 months. The positive effects were not paid off by rebound insomnia [50].

In addition, a systematic review on zolpidem in patients older than 60 years summarized that Zolpidem was effective at reducing SL and thereby increasing TST without significant negative effects [7]. It concluded that zolpidem was well-suited for short-term use, but its long-term effects were still rather unknown, pointing to a poor study quality and high number of methodological flaws.

In contrast, a systematic review on sedative hypnotics published in 2005, which used the age of 60 years to define older people, calculated a number needed to treat for BDLM of 13 with the number needed to harm estimated at 6 for the researched age group [51]. This review concluded that BDLM should be avoided.

Further studies and reviews of case reports and prescription data point to the abuse potential and induction of dependence of BDLM [52, 53]. An examination of 33,240 reports of suspected adverse drug reactions to the European Medicines Agency between 2003 and 2017 established a great risk of dependence as well as a massive potential for abuse with the authors estimating that current data potentially starkly underestimate the real prevalence of BDLM misuse [54].

Due to the heterogeneity in study designs and duration only three case-control studies estimating the effect of Zolpidem on the risk of hip fracture [43, 46, 47] could be included in a meta-analysis. The calculated OR is 1.50 (CI 95 0.96–2.34), where significance is just missed.

The data of two studies estimating the increase in risk of BDLM users versus non-users for all types of fractures, ascertaining the exposure to BDLM using prescription data [41, 44], have been used in a meta-analysis with a resulting OR of 1.22 (CI 95 1.01–1.48), pointing to a significant relationship between BDLM use and an elevation to the risk of fractures.

When considering the evidence provided by the observational studies on associations of BDLM with specific health outcomes, certain limitations apply. The first issue to consider is the method of ascertaining exposure to BDLM; only one study used blood samples to confirm exposure, while the majority relied on prescription data, which do not necessarily translate to exposure with the pharmacological agent.

A further limitation applying to the included case-control studies is that they failed to identify insomnia as a confounding factor for fractures and falls, thereby rendering their results of questionable reliability.

A limitation inherent to the case-crossover study is its inability to measure or evaluate the effect of chronic exposure or use [55].

The cohort study [48] was adjusted for insomnia as a confounding factor by comparing the risk for accidents between groups of patients who were prescribed different hypnotic medications and still found a positive association between the use of BDLM and the risk for accidents.

A prospective cohort study conducted in Norway and published in 2004 [49], which narrowly missed the age inclusion criteria of this systematic review appears to confirm the results on the association between BDLM and hip fractures: it estimated a standardized incidence ratio of 1.2 (CI 95 1.1–1.2).

Moreover, a meta-analysis published in 2017 [56] estimated the association between BDLM and falls, fractures and injuries, using data from patients older than 18 years with an OR of 1.63 (CI 95 1.42–1.87). However, due to the large heterogeneity of studies summarized (*I*^2^ =90%), the reliability of the calculated odds has to be critically scrutinized.

Despite the association between BDLM and an increase in falls and fractures found in these observational studies, neither falls nor fractures feature in the reported adverse events of the included RCTs, which is most likely due to the inherent differences in the study designs. While RCTs tend to exclude patients, who are more at risk of having adverse event and focus on efficacy rather than safety, the included observational studies put the spotlight on previously defined safety aspects of BDLM therapy and established a correlation between BDLM intake and falls and fractures. RCTs also potentially provide insufficient sample size and insufficient study duration to produce data on rare adverse events or adverse events that might develop after longer periods of use.

Concerning our recommendation of discontinuing BDLM in older adults, caution is advised. The process of discontinuation should be conducted as a gradual process in accordance with the respective guidelines for discontinuation of BDZ and BDLM [57].

Within the inclusion criteria of this systematic review, no studies on the association between BDLM and the onset and progress of Alzheimer’s disease or other forms of dementia were found. However, in a recently published review by Ettcheto et al., the authors were convinced to have enough evidence to recommend an extremely cautious attitude towards the use of BDLM in patients with a family history of or suffering from Alzheimer’s disease [58].

### Limitations

This systematic review has several limitations. One of the major limitations is the small number of eligible studies included in the meta-analyses that have a high level of heterogeneity, which diminishes the predictive certainty.

Furthermore, the search strategy and inclusion criteria were designed to identify studies focusing on older people; studies on the general population that may have contained relevant information for the older population might have been overlooked. However, the risk was minimized through examination of the full texts of references where these data were unclear in the abstracts. We also checked the reference lists of all included studies to identify further eligible studies. In addition, using independent reviewers for study selection, evaluation of bias and extraction of data should have minimized this problem. We could also have missed studies because of language bias as we only included studies written in English or German.

Another limitation is the discrepancy in safety data between interventional and observational studies. Due to the limited participant size and duration of the included interventional studies, rare adverse events or adverse events that develop over a certain duration of intake are not reflected in their safety data and the increased risk of falls and fractures that our recommendation is based on can only be found in the observational studies, which have much larger sample sizes and study durations. Finally, our recommendation only focusses on the discontinuation of BDLM. Nevertheless, this systematic review aims at providing an overview on the existing evidence on both the benefits and the risks of the use of BDLM in older people.

## Conclusion

This review underscores the lack of high-quality evidence on the benefits and risks of BDLM treatment for insomnia. In short-term studies, the intake of BDLM appears to improve both sleep and daytime parameters, while producing neither hangover, induction of tolerance nor dependence and virtually no ADE other than unpleasant taste when compared to placebo. However, no long-term controlled prospective studies on the use of BDLM in older people are available. Furthermore, in observational studies, the use of BDLM is associated with a significant increase in the risk for falls and fractures.

Our recommendation is that clinicians should consider discontinuing BDLM, principally because of the high risk for falls and fractures.

High quality and independent studies on the risks and benefits of BDLM use for insomnia in older populations, especially in the light of the lack of long-term studies, are needed in order to enable evidence-based decision making on an individual patient level.

## Supplementary Information

**Additional file 1.** Search Term

**Additional file 2.** List of Excluded Studies

**Additional file 3.** Characteristics of Participants

**Additional file 4.** Findings Controlled Studies

**Additional file 5.** Findings Observational Studies

## Data Availability

The data supporting the conclusions of this article is included within the article and its additional files.

## Abbreviations

BDLM: Benzodiazepine like medications
BDZ: Benzodiazepines
GABA: γ-Aminobutyric acid
ADE: Adverse drug events
PIM: Potentially inappropriate medications
AMSTAR: Critical appraisal tool for systematic reviews
CASP: Critical appraisal skills program
RoB2: Cochrane risk of bias tool
RCT: Randomized-controlled trials
GRADE: Grading of recommendations assessment, development and evaluation
