# Supplementary material for: Efficacy and safety of Z-substances in the management of insomnia in older adults: a systematic review for the development of recommendations to reduce potentially inappropriate prescribing
Source: BMC Geriatr. 2022 Feb 1;22:87. doi: 10.1186/s12877-022-02757-6 (PMC9887772; doi:10.1186/s12877-022-02757-6)
Supplement: Supplementary file 1 — Additional file 1. Search Term [file 12877_2022_2757_MOESM1_ESM.docx]

# Additional file 1: Search Term

| P(1) | Patients |
| --- | --- |
| 1 | geriatrics.mp. or exp geriatrics/ |
| 2 | geriatric patient.mp. |
| 3 | geriatric*.mp. |
| 4 | (elder$ or geriatric$).ab,ti. |
| 5 | elder*.mp. |
| 6 | frail elderly.mp. or exp frail elderly/ |
| 7 | aged.mp. or exp Aged/ |
| 8 | old*.mp. |
| 9 | old* adult*.mp. |
| 10 | old* people*.mp. |
| 11 | >65.mp. |
| 12 | over 65.mp. |
| 13 | or/1-12 |
| P(2) | Condition |
| 14 | insomnia.mp. |
| 15 | disorders of initiating and maintaining sleep.mp or DIMS.mp |
| 16 | awakening, early.mp. or early awakening.mp |
| 17 | nonorganic insomnia.mp. or insomnia, nonorganic.mp |
| 18 | primary insomnia.mp or insomnia, primary.mp |
| 19 | secondary insomnia.mp or insomnia, secondary.mp |
| 20 | transient insomnia.mp or insomnia, transient.mp |
| 21 | sleep initiation dysfunction*.mp or dysfunction*, sleep initiation.mp |
| 22 | rebound insomnia.mp or insomnia, rebound.mp |
| 23 | sleeplessness.mp |
| 24 | insomnia disorder*.mp |
| 25 | insomnia*.mp |
| 26 | chronic Insomnia.mp or insomnia, chronic.mp |
| 27 | psychophysiological insomnia.mp or insomnia, psychophysiological.mp |
| 28 | or/14-27 |
| I | Drugs |
| 29 | Benzodiazepine related drugs.mp |
| 30 | Z-drugs.mp |
| 31 | Z-substances.mp or Z-medication.mp |
| 32 | Eszopiclone.mp |
| 33 | Zaleplon.mp |
| 34 | Zolpidem.mp |
| 35 | Zopiclone.mp |
| 36 | Or/29-35 |
| O | Outcomes |
| 37 | mortality.mp. or exp mortality/ |
| 38 | quality of life.mp. or exp quality of life/ |
| 39 | QOL.mp. |
| 40 | hospitalization.mp. or exp hospitalization/ |
| 41 | hospitalisation.mp. or exp hospitalisation/ |
| 42 | life expectancy.mp. |
| 43 | headache.mp. |
| 44 | diarrhea.mp. |
| 45 | constipation.mp. |
| 46 | cognitive impairment.mp. |
| 47 | cognitive status.mp. |
| 48 | functional status.mp. |
| 49 | functional impairment.mp. |
| 50 | abdominal pain.mp |
| 51 | vertigo.mp |
| 52 | drowsiness.mp |
| 53 | adverse effects.mp. or exp adverse effects/ |
| 54 | drug toxicity.mp. or exp drug toxicity/ |
| 55 | safety.mp. |
| 56 | patient safety.mp. or exp patient safety/ |
| 57 | falls.mp. |
| 58 | delirium.mp. or exp delirium/ |
| 59 | vomiting.mp |
| 60 | nausea.mp |
| 61 | rash.mp |
| 62 | dizziness.mp |
| 63 | hallucinations.mp |
| 64 | cardiovascular.mp |
| 65 | nightmares.mp |
| 66 | renal insufficiency, chronic.mp or kidney failure, chronic.mp |
| 67 | addiction.mp |
| 68 | tolerance.mp |
| 69 | fractures.mp |
| 70 | irritability.mp |
| 71 | confusion.mp or confused.mp |
| 72 | amnesia.mp or anterograde amnesia.mp |
| 73 | accident risk*.mp or accident proneness.mp |
| 74 | or/37-73 |
| S | Studies |
| 75 | Cohort studies.mp |
| 76 | Case-control studies.mp |
| 77 | Randomized controlled trial.mp |
| 78 | Non-randomized controlled trial.mp |
| 79 | Systematic review.mp |
| 80 | Meta-analysis.mp or network meta-analysis.mp |
| 81 | or/75-80 |
| 82 | 13 and 28 and 36 and 74 and 81 |
