# Supplementary material for: Efficacy and safety of Z-substances in the management of insomnia in older adults: a systematic review for the development of recommendations to reduce potentially inappropriate prescribing
Source: BMC Geriatr. 2022 Feb 1;22:87. doi: 10.1186/s12877-022-02757-6 (PMC9887772; doi:10.1186/s12877-022-02757-6)
Supplement: Supplementary file 2 — Additional file 2. List of Excluded Studies [file 12877_2022_2757_MOESM2_ESM.docx]

# Additional file 2: Excluded studies with a reason

| Abad 2018 (1) | Study type |
| --- | --- |
| Allain 1997 (2) | Study type |
| Allain 2003 (3) | Study design |
| Allain 2005 (4) | Study type |
| Ancoli-Israel 2005 (5) | Study design |
| Andrade 2018 (6) | Study type |
| Bain 2006 (7) | Population |
| Bakken 2014 (8) | Population |
| Boyle 2018 (9) | Study design |
| Chung 2013 (10) | Population |
| Cotroneo 2004 (11) | Data not extractable |
| Dehlin 1982 (12) | Study design |
| Dolder 2007 (13) | Population |
| Dombrowsky 2011 (14) | Study type |
| Elie 1983 (15) | Data not extractable |
| Fry 2000 (16) | Population |
| Glass 2005 (17) | Study type |
| Hajak 1998 (18) | Study type |
| Harrison 2005 (19) | Population |
| Hedner 2000 (20) | Population |
| Hosono 2014 (21) | Population |
| Kajiwara 2016 (22) | Study type |
| Kerr 1995 (23) | Study design |
| Kummer 1993 (24) | Study design |
| Lönnroos 2013 (25) | Study design |
| Morishita 2000 (26) | Study type |
| Omvik 2008 (27) | Population |
| Schroeck 2016 (28) | Study type |
| Treves 2018 (29) | Study type |
| Uchimura 2012 (30) | Study design |
| Wilt 2016 (31) | Study type |

# References

1. Abad VC, Guilleminault C. Insomnia in Elderly Patients: Recommendations for Pharmacological Management. Drugs and Aging. 2018;35(9):791-817.

2. Allain H, Monti J. General safety profile of zolpidem: Safety in elderly, overdose and rebound effects. European Psychiatry. 1997;12(SUPPL. 1):21S-9S.

3. Allain H, Bentué-Ferrer D, Tarral A, Gandon JM. Effects on postural oscillation and memory functions of a single dose of zolpidem 5 mg, zopiclone 3.75 mg and lormetazepam 1 mg in elderly healthy subjects. A randomized, cross-over, double-blind study versus placebo. European Journal of Clinical Pharmacology. 2003;59(3):179-88.

4. Allain H, Bentué-Ferrer D, Polard E, Akwa Y, Patat A. Postural instability and consequent falls and hip fractures associated with use of hypnotics in the elderly: a comparative review. Drugs & aging. 2005;22(9):749-65.

5. Ancoli-Israel S. Sleep and aging: Prevalence of disturbed sleep and treatment considerations in older adults. Journal of Clinical Psychiatry. 2005;66(SUPPL. 9):24-30.

6. Andrade C. Sedative hypnotics and the risk of falls and fractures in the elderly. Journal of Clinical Psychiatry. 2018;79(3).

7. Bain KT. Management of chronic insomnia in elderly persons. American Journal Geriatric Pharmacotherapy. 2006;4(2):168-92.

8. Bakken MS, Engeland A, Engesæter LB, Ranhoff AH, Hunskaar S, Ruths S. Risk of hip fracture among older people using anxiolytic and hypnotic drugs: a nationwide prospective cohort study. Eur J Clin Pharmacol. 2014;70(7):873-80.

9. Boyle J, Danjou P, Alexander R, Calder N, Gargano C, Agrawal N, et al. Tolerability, pharmacokinetics and night-time effects on postural sway and critical flicker fusion of gaboxadol and zolpidem in elderly subjects. Br J Clin Pharmacol. 2018;67(2):180-90.

10. Chung SD, Lin CC, Wang LH, Lin HC, Kang JH. Zolpidem Use and the Risk of Injury: A Population-Based Follow-Up Study. PLoS ONE. 2013;8(6).

11. Cotroneo A, Gareri P, Lacava R, Cabodi S. Use of zolpidem in over 75-year-old patients with sleep disorders and comorbidities. Archives of Gerontology and Geriatrics. 2004;38(SUPPL.):93-6.

12. Dehlin O, Rundgren A, Börjesson L, Ekelund P, Gatzinska R, Hedenrud B, et al. Zopiclone to geriatric patients. A parallel double-blind dose-response clinical trial of zopiclone as a hypnotic to geriatric patients - a study in a geriatric hospital. International pharmacopsychiatry. 1982;17 Suppl 2:173-8.

13. Dolder C, Nelson M, McKinsey J. Use of non-benzodiazepine hypnotics in the elderly: Are all agents the same? CNS Drugs. 2007;21(5):389-405.

14. Dombrowsky JW, Lettieri CJ. Eszopiclone in the management of insomnia among elderly patients. Clinical Medicine Insights: Geriatrics. 2011;4:1-8.

15. Elie R, Deschenes JP. Efficacy and tolerance of zopiclone in insomniac geriatric patients. Pharmacology. 1983;27(SUPPL. 2):179-87.

16. Fry JM, Scharf M, Mangano R, Fujimori M, Berkowitz D, Bielksi R, et al. Zaleplon improves sleep without producing rebound effects in outpatients with insomnia. International Clinical Psychopharmacology. 2000;15(3):141-52.

17. Glass J, Lanctôt KL, Herrmann N, Sproule BA, Busto UE. Sedative hypnotics in older people with insomnia: Meta-analysis of risks and benefits. British Medical Journal. 2005;331(7526):1169-73.

18. Hajak G, Bandelow B. Safety and tolerance of zolpidem in the treatment of disturbed sleep: A post-marketing surveillance of 16 944 cases. International Clinical Psychopharmacology. 1998;13(4):157-67.

19. Harrison TS, Keating GM. Zolpidem: A review of its use in the management of insomnia. CNS Drugs. 2005;19(1):65-89.

20. Hedner J, Yaeche R, Emilien G, Farr I, Salinas E. Zaleplon shortens subjective sleep latency and improves subjective sleep quality in elderly patients with insomnia. International Journal of Geriatric Psychiatry. 2000;15(8):704-12.

21. Hosono T, Homma M, Satoh M, Kohda Y. Variables influencing patient satisfaction for hypnotics: Difference between zolpidem and brotizolam. Journal of Clinical Pharmacy and Therapeutics. 2014;39(5):507-10.

22. Kajiwara A, Yamamura M, Murase M, Koda H, Hirota S, Ishizuka T, et al. Safety analysis of zolpidem in elderly subjects 80 years of age or older: adverse event monitoring in Japanese subjects. Aging & mental health. 2016;20(6):611-5.

23. Kerr JS, Dawe RA, Parkin C, Hindmarch I. Zopiclone in elderly patients: Efficacy and safety. Human Psychopharmacology. 1995;10(3):221-9.

24. Kummer J, Guendel L, Linden J, Eich FX, Attali P, Coquelin JP, et al. Long-term polysomnographic study of the efficacy and safety of zolpidem in elderly psychiatric in-patients with insomnia. Journal of International Medical Research. 1993;21(4):171-84.

25. Lönnroos E, Dawson J, Ilomäki J, Bell JS. Nonbenzodiazepine hypnotics are associated with an increased risk of hip fracture in residential aged care facilities. Aging Health. 2013;9(4):421-3.

26. Morishita S, Sonohara M, Murakami H, Yoshida S, Aoki S. Long-term treatment of brotizolam and zopiclone in elderly insomniacs. Kawasaki Medical Journal. 2000;26(1):9-11.

27. Omvik S, Sivertsen B, Pallesen S, Bjorvatn B, Havik OE, Nordhus IH. Daytime functioning in older patients suffering from chronic insomnia: Treatment outcome in a randomized controlled trial comparing CBT with Zopiclone. Behaviour Research and Therapy. 2008;46(5):623-41.

28. Schroeck JL, Ford J, Conway EL, Kurtzhalts KE, Gee ME, Vollmer KA, et al. Review of Safety and Efficacy of Sleep Medicines in Older Adults. Clin Ther. 2016;38(11):2340-72.

29. Treves N, Perlman A, Geron LK, Asaly A, Matok I. Z-drugs and risk for falls and fractures in older adults-a systematic review and meta-analysis. Age and Ageing. 2018;47(2):201-8.

30. Uchimura N, Kamijo A, Takase T. Effects of eszopiclone on safety, subjective measures of efficacy, and quality of life in elderly and nonelderly Japanese patients with chronic insomnia, both with and without comorbid psychiatric disorders: a 24-week, randomized, double-blind study. Annals of General Psychiatry. 2012;11.

31. Wilt TJ, MacDonald R, Brasure M, Olson CM, Carlyle M, Fuchs E, et al. Pharmacologic treatment of insomnia disorder: An evidence report for a clinical practice guideline by the American college of physicians. Annals of Internal Medicine. 2016;165(2):103-12.
