# Supplementary material for: Efficacy and safety of Z-substances in the management of insomnia in older adults: a systematic review for the development of recommendations to reduce potentially inappropriate prescribing
Source: BMC Geriatr. 2022 Feb 1;22:87. doi: 10.1186/s12877-022-02757-6 (PMC9887772; doi:10.1186/s12877-022-02757-6)
Supplement: Supplementary file 3 — Additional file 3. Characteristics of Participants [file 12877_2022_2757_MOESM3_ESM.docx]

**Additional file 3: Study participants**

| Reference | General framework/ country/ ethnicity | Age in  Years (SD) | Male Gender | Comorbidities | Co-medication | Physical examination | Cognitive examination |
| --- | --- | --- | --- | --- | --- | --- | --- |
| Ancoli-Israel 2010 (66) | 82 private-practice clinics and clinical research sites  USA  Oct 2006–Feb 2008  White:  Eszopiclone 91.8%  placebo  93.3% | Eszopiclone 71.6 (5.0)  placebo  72.4 (5.2) | Eszopiclone 36.1%  placebo 38.7% | Comorbid conditions by organ system  Eszo 2mg n (%), placebo n (%):  Musculoskeletal  125 (64.4), 126 (64.9)  Endocrine and metabolic  123 (63.4), 118 (60.8)  Cardiovascular  118 (60.8), 117 (60.3)  Gastrointestinal  89 (45.9), 88 (45.4)  Genitourinary  51 (26.3), 45 (23.2) Neurologic  47 (24.2), 34 (17.5)  Psychiatric/Psychological  17 (8.8), 18 (9.3) | Concomitant medications by ATC level  Eszo 2mg n (%), placebo n (%):  Lipid modifying agents  89 (45.9), 87 (44.8)  Antithrombotic agents  62 (32.0), 66 (34.0)  Anti-inflammatory &  antirheumatic products  58 (29.9), 61 (31.4)  Agents acting on the renin-angiotensin system  59 (30.4), 59 (30.4)  Analgesics  37 (19.1), 52 (26.8)  Mineral supplements  42 (21.6), 45 (23.2)  Psychoanaleptics  7 (3.6), 5 (2.6)  Psycholeptics  7 (3.6), 7 (3.6) | Weight  BMI | Mini-Mental State Examination score $\geq28$ |
| Ancoli-Israel 1999 (67) | 35 centers  USA  White:  Zale5 94.6%  Zale10 93.9%  Zolpi 94.6%  placebo 90.7% | Zale5  71.5 (4.8)  Zale10  71.6 (5.7)  Zolpi  72.1 (5.2)  placebo  71.6 (5.3) | Zale5 42.2%  Zale10 42.4%  Zolpi  43.2%  placebo 40.2% | No information | No information | Weight  Sitting blood pressure  Pulse rate  Body temperature | Zung anxiety and Zung depression score <50 |
| Avidan 2010 (83) | Retrospective cohort study using Thomson MarketScan® Medicare Supplemental and Coordination of Benefits data bases  January 1^st^ 2000-June 30^th^ 2006, USA  No information | BDLM 75.1 (7.0) MRA 75.4 (7.2) | BDLM 39.8% MRA 37.8% | Comorbidity  22,507 (14,34)  Adjustment disorders and stress  417 (0.27)  Psychotic disorder  1,379 (0.88)  Mood disorder  5,527 (3.52)  Anxiety  2,023 (1.29)  Other mental health disorder  5,529 (3.52)  Alcohol or drug abuse  4,379 (2,79)  Seizure disorder  2,206 (1.41)  Abnormal movement or Parkinson  5,828 (3.71)  Headache  3,520 (2.24)  Pain  10,538 (6.71) | Concomitant medication  84,048 (53.54)  Antihistamines  15,229 (9.70)  Anticholinergics/antiparkinsonians  /antispastics  3,169 (2.02)  Opiate antagonists  7 (0.00)  Analgesics  38,844 (24.74)  Antidiabetics  20,906 (13.32)  Antimanic  290 (0.18)  Anxiolytic  3,426 (2.18)  Antidepressants  27,125 (17,28)  Benzodiazepines  13,904 (8.86)  Anticonvulsants  10,357 (6.60) | No information | No information |
| Berry 2013 (74) | Case-crossover study using Medicare part A and D claims  July 1^st^ 2007 - December 31^st^ 2008  USA  White: 89.7% | 81.0 (9.7) | 22.4% | Anemia  6.8%  Rheumatoid arthritis (RA)  8.9%  Congestive heart failure  20.7%  Depression  49.9%  Diabetes mellitus (DM)  31.7%  Stroke  15.2% | No information | No information | Normal or mild impairment  60.4%  moderate to severe  39.6% |
| Chang 2011 (75) | Case-control study using Patient- Safety Reporting System at Chang Gung Memorial Hospital, Linko  January 1^st^ 2006 - December 31^st^ 2006  Taiwan  Taiwanese | All subjects ≥65 | No information | No information | No information | No information | No information |
| Dehlin 1995 (68) | 10 geriatric clinics  Sweden  No information | Mean (range)  79(60-95)  No significant difference between groups | Zopi 26%  Fluni 32.7% | Stroke  Z=11 F=14  Other neurological  Z=2 F=4  Fracture  Z=7 F=4  DM  Z=3 F=1  Other disease  Z=27 F=29 | Bronchodilators, diuretics, beta-blockers, analgesics, antihistamines evenly distributed between groups | Weight | MMSE  Z 27.5 (2.5)  F 27.8 (2.6) |
| Elie 1990 (69) | Residential homes  Canada  No information | 76.0 (1.3) | 25% | No information | Medication without CNS effects or interactions with experimental drugs permitted, supportive chloral hydrate 500mg authorised PRN on Friday, Saturday and Sunday nights | No information | No information |
| Kang 2012 (76) | Case-crossover study using Health Insurance Review and Assessment Service database  Jan 2005-Jun 2006  South Korea  No information | All subjects ≥65 | 20.0% | Comorbidity before fracture n (%)  Hypertension 763 (50.6)  Osteoarthritis 627 (41.6)  Osteoporosis 469 (31.1)  DM 432 (28.6)  Anemia 326 (21.6)  Depression 242 (16.0)  Dementia 147 (9.7)  Stroke 134 (8.9)  RA 82 (5.4)  Heart failure 80 (5.3)  Visual disturbance 41 (2.7)  Parkinson’s disease 33 (2.2) | Conditional logistic regression analysis of antidepressants, anxiolytics, diuretics, alpha blockers, beta blockers, and vasodilators did not have a significant effect on fracture on the study data. Thus, they were excluded from the final analysis model. | No information | No information |
| Klimm 1987 (70) | Community for old age, Germany  No information | 73.2 (1.5) | 19.2% | 70/72 had concomitant diseases, most frequently arthritis, circulatory disorders, hypertension, and cardiac insufficiency | All patients continued to receive their regular medication provided that it was initiated >1 week before the first consultation of the study | Standing systolic and diastolic blood pressure | Intellectual Quotient and Syndrom Kurztest normal range for age |
| Lai 2015 (77) | Case–control study using Taiwan National Health Insurance Program data  1998- 2011  Taiwan  Taiwanese | Cases:  77.0 (7.4)  Controls:  74.1 (6.5) | Cases:  40.1%  Controls:  40.0% | Comorbidities before index date  Cases vs controls n (%)  Alcohol-related disease  8 (0.1) vs 3 (<0.1)  Cancer  528 (4.8) vs 352 (3.9)  Cardiovascular disease  5,711 (51.9) vs 4,915 (54.1)  Chronic kidney disease  585 (5.3) vs 400 (4.4)  Chronic obstructive pulmonary disease  3,078 (28.0) vs 2,593 (28.6)  DM  2,750 (25.0) vs 2,236 (24.6)  Dementia  769 (7.0) vs 449 (4.9)  Depression  456 (4.1) vs 254 (2.8)  Hyperlipidemia  2,969 (27.0) vs 2,517 (27.7)  Hypertension  7,572 (68.9) vs 6,350 (69.9)  Osteoporosis  2,673 (24.3) vs 2,183 (24.0)  Parkinson’s disease  346 (3.1) vs 160 (1.8) | Number of medications  Cases vs controls mean (SD)  3.1 (3.1) vs 2.9 (2.9) | No information | No information |
| Leppik 1997 (71) | Multicenter outpatient study  USA  White:  93% | Mean (range)  69(59-85) | 37% | No information | No information | Weight:  mean 74.3kg (range 39-134)  ECG  Lab evaluation | No information |
| Pierfitte 2001 (78) | Case-control study using emergency department data of Pellegrin and Saint André university hospitals, Bordeaux  January 1996- July 1997  France  No information | All subjects ≥65 | No information | No information | No information | BMI | No information |
| Roger 1993 (72) | Multicenter inpatient study on 7 geriatric wards, France and Belgium  No information | Zolpi 5mg 81.2 (1.1)  Zolpi 10mg 79.8 (0.8)  Tria 0.25mg 82.2 (0.8) | Zolpi 5mg 27.1%  Zolpi 10mg 25.7%  Tria 0.25mg 24.7% | No information | No information | Weight  Height | No information |
| Scharf 2005 (73) | Multicenter outpatient study  USA  White:  Eszo 1mg 95.8%  Eszo 2mg 100%  placebo 93.8% | Eszo 1mg 72.7 (4.5)  Eszo 2mg 72.2 (5.3)  placebo 72.0 (5.0) | Eszo 1mg 43.1%  Eszo 2mg 45.6%  placebo 38.8% | Comorbid conditions  Eszo 1mg % of n=72  Eszo 2mg % of n=79  placebo % of n=80:  Any comorbid condition 96, 96, 99  Hypertension 31, 23, 23  Hypercholesterolemia 17, 14, 18  Hypothyroidism 10, 11, 13  Osteoarthritis 11, 6, 13  Presbyopia 10, 13, 13 | Concomitant medication  Eszo 1mg % of n=72, Eszo 2mg % of n= 79, placebo % of n=80:  Concomitant medication 92, 89, 94  Aspirin 22, 19, 23  Estrogen 18, 8, 16  Calcium 14, 13, 15  Levothyroxine 7, 14, 13  Acetaminophen 8, 9, 11 | Weight  BMI | Physical and brief neurological examination |
| Tang 2015 (79) | Case-crossover study using the Longitudinal Health Insurance Database  2002-2010  Taiwan  No information | 65-74  n=2482  75-84  n=2809  ≥85  n=759 | 32.3% | Comorbidity n (%)  Hypertension  3726 (62.0%)  Osteoarthritis  3226 (53.7%)  Osteoporosis  937 (15.6%)  DM  1522 (25.3%)  Anemia  524 (8.7%)  Depression  583 (9.7%)  Dementia  325 (5.4%)  RA  176 (2.9%)  Heart failure  570 (9.5%)  Parkinson’s Disease  318 (5.4%)  Non-stroke CVD  385 (6.4%)  COPD  563 (9.4%)  Chronic renal failure  353 (5.9%)  BPH  831 (13.8%)  Hyperlipidemia  1419 (23.6%)  Gout  734 (12.2%) | No information | No information | No information |
| Tom 2016 (80) | Case-crossover study using Medicare data  2007-2009  USA  White:  TBI: BDLM 92%  Hip fracture: BDLM 93% | TBI BDLM: 80.4 (8.2)  Hip fracture BDLM: 81.9 (8.0) | TBI BDLM: 30%  Hip fracture BDLM: 16% | History of comorbid disease n (%)  TBI:  Alzheimer’s and related dementias  165 (34%)  Atrial fibrillation  169 (34%)  Chronic kidney disease  174 (35%)  COPD  217 (44%)  Depression history  293 (60%)  DM  231 (47%)  Heart failure  303 (62%)  Ischemic heart disease  99 (20%)  Neurologic disease  95 (19%)  Stroke/TIA  166 (34%)  Hip fracture:  Alzheimer’s and related dementias  503 (42%)  Atrial fibrillation  320 (27%)  Chronic kidney disease  376 (32%)  COPD  530 (44%)  Depression history  707 (59%)  DM  499 (42%)  Heart failure  696 (58%)  Ischemic heart disease  320 (27%)  Liver disease  98 (8%)  Neurologic disease  173 (15%)  Stroke/TIA  377 (32%) | Concomitant use of sleep medication (sedating antidepressants, barbiturates, benzodiazepines, chloral hydrate, hydroxyzine, ramelteon)  TBI 23%  Hip fracture 20% | No information | No information |
| Wang 2001 (81) | Case-control study using New Jersey Medicaid Program Database  Jan 1^st^ 1994- Dec 31^st^ 1994  USA  White:  Cases: 90.3%  Controls: 84.5% | Cases: 82.3  Controls: 82.4  All subjects ≥65 | Cases: 16.2%  Controls: 16.4% | Comorbidity index score mean:  Cases 1.3  Controls 0.9  Hospital days prior 6 months mean:  Cases 5.6  Controls 2.8  Outpatient services prior 6 months mean:  Cases 8.3  Controls 7.0  Nursing home stay prior 6 months:  Cases 32.1%  Controls 19.6% | Number of medications mean:  Cases 7.1  Controls 6.2 | No information | No information |
| Zint 2010 (82) | Case–control study using Medicare and Pennsylvania drug assistance program (PACE) data  1994- 2005  USA  No information | All subjects ≥65 | No information | No information | No information | No information | No information |
