# Supplementary material for: Efficacy and safety of Z-substances in the management of insomnia in older adults: a systematic review for the development of recommendations to reduce potentially inappropriate prescribing
Source: BMC Geriatr. 2022 Feb 1;22:87. doi: 10.1186/s12877-022-02757-6 (PMC9887772; doi:10.1186/s12877-022-02757-6)
Supplement: Supplementary file 4 — Additional file 4. Findings Controlled Studies [file 12877_2022_2757_MOESM4_ESM.docx]

**Additional file 4: Findings: controlled studies**

| Reference | Study medication/ comparator | Outcomes | RR, OR, HR | Means or mean differences | Events | P |
| --- | --- | --- | --- | --- | --- | --- |
| Ancoli-Israel 2010 (66) | Eszopiclone 2 mg nightly vs placebo | Adverse events overall Eszo vs placebo |  |  | 50.5% vs 59.3% |  |
|  |  | Headache Eszo vs placebo |  |  | 13.9% vs 12.4% |  |
|  |  | Anxiety Eszo vs placebo |  |  | 0.5% vs 0% |  |
|  |  | Hallucinations Eszo vs placebo |  |  | 1.0% vs 0% |  |
|  |  | Memory impairment Eszo vs placebo |  |  | 0.5% vs 0% |  |
|  |  | Attention disturbance Eszo vs placebo |  |  | 1.5% vs 0% |  |
|  |  | Nervousness Eszo vs placebo |  |  | 2.1% vs 1.0% |  |
|  |  | Nasopharyngitis Eszo vs placebo |  |  | 5.7% vs 6.2% |  |
|  |  | Dizziness Eszo vs placebo |  |  | 4.1% vs 1.5% |  |
|  |  | Falls Eszo vs placebo |  |  | 1.0% vs 0.5% |  |
|  |  | Unpleasant taste Eszo vs placebo |  |  | 12.4% vs 1.5% | p<0.001 |
|  |  | Suicide Eszo vs placebo |  |  | 1 vs 0 |  |
|  |  | Death due to arteriosclerotic heart disease Eszo vs placebo |  |  | 1 vs 0 |  |
|  |  | Cholezystitis Eszo vs placebo |  |  | 1 vs 0 |  |
|  |  | Rectozele Eszo vs placebo |  |  | 1 vs 0 |  |
|  |  | Perforated appendix Eszo vs placebo |  |  | 0 vs 1 |  |
|  |  | Pneumonia Eszo vs placebo |  |  | 0 vs 1 |  |
|  |  | sSL Mean decrease over double-blind period Eszo vs placebo |  | Mean (SD)  19.92 vs 24.62 min  (SD n.r.) |  | p=0.0014 |
|  |  | Daytime alertness mean change on 11-point Likert scale from baseline during double-blind phase: Eszo vs placebo |  | Mean (SD) 1.0 (1.3) vs 0.6 (1.3) |  | p<0.001 |
|  |  | Ability to concentrate mean change on 11-point Likert scale from baseline during double-blind phase: Eszo vs placebo |  | Mean (SD) 1.0 (1.3) vs 0.5 (1.3) |  | p<0.001 |
|  |  | Ability to function mean change on 11-point Likert scale from baseline during double-blind phase: Eszo vs placebo |  | Mean (SD) 0.9 (1.3) vs 0.5 (1.3) |  | p<0.001 |
|  |  | Physical well-being mean change on 11-point Likert scale from baseline during double-blind phase: Eszo vs placebo |  | Mean (SD) 0.9 (1.3) vs 0.5 (1.3) |  | p<0.001 |
|  |  | Quality of life (SF-36) at week 12: Eszo vs placebo |  | Mean (SD) |  |  |
|  |  | Physical functioning score |  | 73.1 (23.0) vs 71.3 (25.8) |  | p=0.99 |
|  |  | Role physical |  | 73.3 (23.3) vs 70.0 (22.6) |  | p=0.23 |
|  |  | Bodily pain |  | 73.0 (21.3) vs 69.8 (23.6) |  | p=0.15 |
|  |  | General health |  | 71.6 (18.9) vs 68.5 (20.1) |  | p=0.009 |
|  |  | Vitality |  | 58.9 (21.2) vs 55.1 (20.3) |  | p=0.008 |
|  |  | Social functioning |  | 78.7 (20.9) vs 79.9 (21.4) |  | p=0.74 |
|  |  | Role emotional |  | 79.1 (21.5) vs 77.0 (22.0) |  | p=0.20 |
|  |  | Mental health |  | 75.5 (16.4) vs 73.9 (18.4) |  | p=0.07 |
| Ancoli-Israel 1999 (67) | Zaleplon 5 mg, Zaleplon 10 mg, Zolpidem 5 mg, placebo | Adverse Events: overall (headache, pain, somnolence, rhinitis)  Zale 5 vs Zale 10 vs Zolpidem vs placebo |  |  | 56% vs 59% vs 63% vs 56% |  |
|  |  | ZNS adverse events Zolpidem vs placebo |  |  | 25% vs 14% | p<0.05 |
|  |  | Somnolence Zale 5 vs Zolpidem vs placebo |  |  | 4% vs 10% vs 2% | p<0.05 |
|  |  | Improvement of sleep quality on a 1-7 Likert scale relative to baseline week 1 Zale 5 vs placebo | OR (CI95) 1.34 (0.82-2.19) |  |  | Difference to placebo: p=0.239 |
|  |  | Improvement of sleep quality on a 1-7 Likert scale relative to baseline week 1 Zale 10 vs placebo | OR (CI95) 1.12 (0.69-1.82) |  |  | Difference to placebo:  p=0.653 |
|  |  | Improvement of sleep quality on a 1-7 Likert scale relative to baseline week 1 Zolpi 5 vs placebo | OR (CI95) 3.16 (1.78-5.64) |  |  | Difference to placebo:  p<0.001 |
|  |  | Improvement of sleep quality on a 1-7 Likert scale relative to baseline week 2 Zale 5 vs placebo | OR (CI95) 0.79 (0.48-1.30) |  |  | Difference to placebo:  p=0.358 |
|  |  | Improvement of sleep quality on a 1-7 Likert scale relative to baseline week 2 Zale 10 vs placebo | OR (CI95) 0.86 (0.52-1.42) |  |  | Difference to placebo:  p=0.560 |
|  |  | Improvement of sleep quality on a 1-7 Likert scale relative to baseline week 2 Zolpi 5 vs placebo | OR (CI95) 1.50 (0.86-2.63) |  |  | Difference to placebo:  p=0.152 |
| Dehlin 1995 (68) | Zopiclone 5 mg and Flunitrazepam 1 mg | Week 2: sleep quality on VAS Zopi vs Fluni |  | 30.7 (15.8) v. 27.0 (20.6) |  | p=0.08 |
|  |  | Week 2: number of awakenings per night Zopi vs Fluni |  | 1.36 (0.7) vs 1.38 (1.0) |  | p=0.36 |
|  |  | Week 2: feeling upon awakening on VAS Zopi vs Fluni |  | 31.4 (18.4) vs 33.2 (22.6) |  | p=0.65 |
|  |  | Week 2: ease of awakening on VAS Zopi vs Fluni |  | 42.3 (19.1) vs 45.4 (25.0) |  | p=0.73 |
|  |  | Week 2: feeling of rest on VAS Zopi vs Fluni |  | 38.4 (19.5) vs 44.3 (22.9) |  | p=0.38 |
|  |  | Week 2: difficulty falling asleep on VAS Zopi vs Fluni |  | 28.9 (19.4) vs 23.2 (17.2) |  | p=0.04 |
|  |  | Week2: remembering dreams on VAS Zopi vs Fluni |  | 75.9 (20.0) vs 71.9 (24.9) |  | p=0.22 |
|  |  | Week 2: state of calmness on VAS Zopi vs Fluni |  | 29.8 (19.6) vs 31.4 (24.8) |  | p=0.42 |
|  |  | Week 2: daytime alertness on VAS Zopi vs Fluni |  | 31.7 (20.4) vs 33.0 (23.1) |  | p=0.55 |
|  |  | Week 2: number for daytime naps Zopi vs Fluni |  | 2.8 (0.7) vs 2.9 (0.7) |  | p=0.87 |
|  |  | Week 3: sleep quality on VAS Zopi vs Fluni |  | 35.5 (21.4) vs 30.8 (20.2) |  | p=0.09 |
|  |  | Week 3: number of awakenings Zopi vs Fluni |  | 1.58 (0.92) vs 1.49 (1.17) |  | p=0.08 |
|  |  | Week 3: feeling upon awakening on VAS Zopi vs Fluni |  | 32.2 (21.2) vs 29.6 (19.0) |  | p=0.78 |
|  |  | Week 3: ease of awakening on VAS Zopi vs Fluni |  | 44.1 (23.5) vs 41.7 (20.5) |  | p=0.40 |
|  |  | Week 3: feeling of rest on VAS Zopi vs Fluni |  | 40.2 (22.8) vs 40.5 (20.4) |  | p=0.93 |
|  |  | Week 3: difficulty falling asleep on VAS Zopi vs Fluni |  | 34.6 (22.7) vs 24.2 (18.5) |  | p=0.002 |
|  |  | Week 3: remembering dreams on VAS Zopi vs Fluni |  | 76.1 (22.5) vs 70.9 (25.3) |  | p=0.55 |
|  |  | Week 3: state of calmness on VAS Zopi vs Fluni |  | 33.1 (22.8) vs 30.1 (23.0) |  | p=0.97 |
|  |  | Week 3: daytime alertness on VAS Zopi vs Fluni |  | 33.8 (22.6) vs 32.1 (23.0) |  | p=0.69 |
|  |  | Week 3: number for daytime naps Zopi vs Fluni |  | 2.9 (0.7) vs 2.7 (0.7) |  | p=0.44 |
|  |  | Number of AEs week 1 single-blind placebo Zopi vs Fluni |  |  | 23 vs 34 |  |
|  |  | Number of AEs week 2 active treatment Zopi vs Fluni |  |  | 24 vs 30 |  |
|  |  | Number of AEs week 3 active treatment Zopi vs Fluni |  |  | 23 vs 23 |  |
|  |  | Number of AEs week 4 single-blind placebo Zopi vs Fluni |  |  | 15 vs 18 |  |
|  |  | Vertigo active treatment Zopi vs Fluni |  |  | 10 vs 4 |  |
|  |  | Depression active treatment Zopi vs Fluni |  |  | 7 vs 10 |  |
|  |  | Arthralgia active treatment Zopi vs Fluni |  |  | 7 vs 5 |  |
|  |  | Diarrhea active treatment Zopi vs Fluni |  |  | 2 vs 6 |  |
|  |  | Headache active treatment Zopi vs Fluni |  |  | 2 vs 6 |  |
| Elie 1990 (69) | Zopiclone 5/7.5 mg, Triazolam 0.125/0.25 mg or placebo | Reduction of dreams Zopi vs Tria vs placebo |  |  | 5 vs 3 vs 0 | Zopi to placebo p<0.02 |
|  |  | Bitter taste Zopi vs Tria vs placebo |  |  | 5 vs 0 vs 1 | Zopi to placebo p<0.06 |
|  |  | Sleep latency on a 10-point Likert scale Zopiclone vs Triazolam vs placebo baseline |  | 4.1 (0.68) vs 3.6 (0.49) vs 4.3 (0.57) |  |  |
|  |  | Sleep latency on a 10-point Likert scale Zopiclone vs Triazolam vs placebo average Rx effect |  | 6.7 (0.07) vs 6.8 (0.07) vs 5.6 (0.05) |  | p<0.05 to placebo |
|  |  | Sleep latency on a 10-point Likert scale Zopiclone vs Triazolam vs placebo average follow-up |  | 4.7 (0.42) vs 4.1 (0.38) vs 5.6 (0.44) |  | p<0.05 to placebo Tria only |
|  |  | Sleep soundness on a 10-point Likert scale Zopiclone vs Triazolam vs placebo baseline |  | 3.3 (0.41) vs 3.3 (0.38) vs 4.1 (0.36) |  |  |
|  |  | Sleep soundness on a 10-point Likert scale Zopiclone vs Triazolam vs placebo average Rx effect |  | 6.8 (0.05) vs 6.4 (0.06) vs 5.7 (0.07) |  | p<0.01 Z to P  p<0.08 T to P |
|  |  | Sleep soundness on a 10-point Likert scale Zopiclone vs Triazolam vs placebo follow up |  | 4.5 (0.38) vs 3.8 (0.28) vs 5.5 (0.42) |  | p<0.08 Z to P  p<0.01 T to P |
|  |  | Quality of sleep on a 10-point Likert scale Zopiclone vs Triazolam vs placebo |  | 7.4 (0.79) vs 8.0 (0.75) vs 7.9 (0.49) |  |  |
|  |  | Quality of sleep on a 10-point Likert scale Zopiclone vs Triazolam vs placebo average Rx effect |  | 10.8 (0.08) vs 11.0 (0.06) vs 10.0 (0.07) |  | p<0.08 Z to P  p<0.08 T to P |
|  |  | Quality of sleep on a 10-point Likert scale Zopiclone vs Triazolam vs placebo follow up |  | 9.0 (0.45) vs 8.2 (0.54) vs 9.9 (0.50) |  | p<0.05 T to P |
|  |  | Morning wake up on a 10-point Likert scale Zopiclone vs Triazolam vs placebo |  | 8.4 (0.78) vs 9.1 (0.58) vs 9.0 (0.70) |  |  |
|  |  | Morning wake up on a 10-point Likert scale Zopiclone vs Triazolam vs placebo average Rx effect |  | 10.5 (0.10) vs 10.5 (0.08) vs 9.9 (0.11) |  |  |
|  |  | Morning wake up on a 10-point Likert scale Zopiclone vs Triazolam vs placebo follow up |  | 8.9 (0.58) vs 9.0 (0.47) vs 10.0 (0.60) |  |  |
|  |  | Hangover on a 10-point Likert scale Zopiclone vs Triazolam vs placebo |  | 15.8 (0.36) vs 15.3 (0.53) vs 15.6 (0.48) |  |  |
|  |  | Hangover on a 10-point Likert scale Zopiclone vs Triazolam vs placebo average Rx effect |  | 16.6 (0.06) vs 16.7 (0.04) vs 16.3 (0.06) |  |  |
|  |  | Hangover on a 10-point Likert scale Zopiclone vs Triazolam vs placebo follow up |  | 16.0 (0.42) vs 16.2 (0.31) vs 16.5 (0.39) |  |  |
|  |  | Changes from baseline: sleep latency on a 10-point Likert scale week 1 Zopiclone vs Triazolam vs placebo |  | 2.8 (0.66) vs 2.8 (0.52) vs 1.5 (0.53) |  |  |
|  |  | Changes from baseline: sleep latency on a 10-point Likert scale week 2 Zopiclone vs Triazolam vs placebo |  | 2.5 (0.71) vs 3.3 (0.56) vs 1.3 (0.63) |  | p<0.05 T to P |
|  |  | Changes from baseline: sleep latency on a 10-point Likert scale week 3 Zopiclone vs Triazolam vs placebo |  | 2.5 (0.75) vs 3.1 (0.63) vs 1.3 (0.65) |  |  |
|  |  | Changes from baseline: sleep soundnes on a 10-point Likert scales week 1 Zopiclone vs Triazolam vs placebo |  | 3.4 (0.50) vs 3.0 (0.50) vs 1.3 (0.34) |  | p<0.01 Z to P  p<0.05 T to P |
|  |  | Changes from baseline: sleep soundness on a 10-point Likert scale week 2 Zopiclone vs Triazolam vs placebo |  | 3.6 (0.50) vs 3.5 (0.48) vs 1.7 (0.39) |  | p<0.01 Z to P  p<0.05 T to P |
|  |  | Changes from baseline: sleep soundness on a 10-point Likert scale week 3 Zopiclone vs Triazolam vs placebo |  | 3.7 (0.52) vs 2.9 (0.54) vs 1.8 (0.40) |  | p<0.01 Z to P |
|  |  | Changes from baseline during withdrawal sleep latency on a 10-point Likert scale day 1 Zopiclone vs Triazolam vs placebo |  | -0.27 vs -0.29 vs 1.53 |  |  |
|  |  | Changes from baseline during withdrawal sleep latency on a 10-point Likert scale day 2 Zopiclone vs Triazolam vs placebo |  | 1.33 vs 0.21 vs 1.67 |  |  |
|  |  | Changes from baseline during withdrawal sleep latency on a 10-point Likert scale day 3 Zopiclone vs Triazolam vs placebo |  | 0.00 vs 0.86 vs 1.40 |  |  |
|  |  | Changes from baseline during withdrawal sleep latency on a 10-point Likert scale day 4 Zopiclone vs Triazolam vs placebo |  | 1.13 vs 1.07 vs 0.60 |  |  |
|  |  | Changes from baseline during withdrawal sleep soundness on a 10-point Likert scale day 1 Zopiclone vs Triazolam vs placebo |  | 0.07 vs -0.57 vs 1.53 |  | p<0.05 Z to P  p<0.01 T to P |
|  |  | Changes from baseline during withdrawal sleep soundness on a 10-point Likert scale day 2 Zopiclone vs Triazolam vs placebo |  | 2.13 vs 0.86 vs 1.67 |  |  |
|  |  | Changes from baseline during withdrawal sleep soundness on a 10-point Likert scale day 3 Zopiclone vs Triazolam vs placebo |  | 1.00 vs 0.36 vs 1.87 |  |  |
|  |  | Changes from baseline during withdrawal sleep soundness on a 10-point Likert scale day 4 Zopiclone vs Triazolam vs placebo |  | 1.6 vs 1.43 vs 0.8 |  |  |
| Klimm 1987 (70) | Zopiclone 7.5 mg or Nitrazepam 5 mg | Sleep latency 0=very fast- 100=very slow Zopi vs Nitra first day active treatment- last day of placebo |  | -18.2 (48.3) vs -15.6 (49.5) |  | p<0.04 Zopi |
|  |  | Quality of sleep 0=very bad- 100= very good Zopi vs Nitra first day active treatment- last day of placebo |  | 24 (45.6) vs 23.1 (37.8) |  | p<0.006 Zopi  p<0.002 Nitra |
|  |  | Feeling on awakening 0=very sleepy-100=very alert Zopi vs Nitra first day active treatment- last day of placebo |  | -5.7 (48.5) vs  -6.8 (49.4) |  |  |
|  |  | Dry mouth P vs Z vs N |  |  | 1 vs 0 vs 0 |  |
|  |  | Bitter taste P vs Z vs N |  |  | 2 vs 1 vs 0 |  |
|  |  | Dizziness P vs Z vs N |  |  | 0 vs 1 vs 0 |  |
|  |  | Confusion P vs Z vs N |  |  | 0 vs 0 vs 1 |  |
|  |  | Fatigue P vs Z vs N |  |  | 0 vs 0 vs 1 |  |
|  |  | Gastralgia P vs Z vs N |  |  | 3 vs 0 vs 0 |  |
|  |  | Sleep disturbance P vs Z vs N |  |  | 7 vs 0 vs 0 |  |
|  |  | Patient withdrawal due to confusion Z vs N |  |  | 0 vs 1 |  |
| Leppik 1997 (71) | Zolpidem 5 mg, Triazolam 0.125 mg, Temazepam 15 mg and placebo | Sleep latency in minutes Zolpi n=82 Tria n=85 Tema n=84 placebo n=84 at baseline |  | 78.1 (5.2) vs 84.7 (5.9) vs 74.1 (4.9) vs 76.9 (5.4) |  | Prop haz model p=0.603 |
|  |  | Sleep latency in minutes Zolpi n=82 Tria n=85 Tema n=83 placebo n=83 at week 1 |  | 44.7 (3.0) vs 60.8 (5.0) vs 43.1 (3.2) vs 63.4 (4.7) |  | Prop haz model p=<.001  p<0.05 Tema to P |
|  |  | Sleep latency in minutes Zolpi n=82 Tria n=85 Tema n=83 placebo n=83 at week 1 mean difference |  | -32.7 (3.9) vs  -24.6 (4.7) vs  -31.0 (4.3) vs  -14.3 (3.9) |  | Prop haz model p=0.004  p<0.05 Z to P  p<0.05 Tema to P |
|  |  | Sleep latency in minutes Zolpi n=79 Tria n=79 Tema n=77 placebo n=79 at week 2 |  | 42.8 (2.9) vs 52.4 (4.1) vs 45.0 (4.0) vs 59.1 (4.4) |  | Prop haz model p=0.036  p<0.05 Z to P |
|  |  | Sleep latency in minutes Zolpi n=79 Tria n=79 Tema n=77 placebo n=79 at week 2 mean difference |  | -36.3 (4.3) vs  -35.4 (4.8) vs  -30.0 (4.5) vs  -19.3 (4.3) |  | Prop haz model p=0.007  p<0.05 Z to P  p<0.05 Tria to P  p<0.05 Tema to P |
|  |  | Sleep latency in minutes Zolpi n=78 Tria n=78 Tema n=77 placebo n=78 at week 3 |  | 43.5 (3.9) vs 56.6 (5.0) vs 39.1 (3.3) vs 79.8 (5.0) |  | Prop haz model p<0.001  p<0.05 Z to P  p<0.05 Tema to P |
|  |  | Sleep latency in minutes Zolpi n=78 Tria n=78 Tema n=77 placebo n=78 at week 3 mean difference |  | -35.8 (4.6) vs  -31.8 (5.3) vs  -35.5 (4.9) vs  -19.5 (4.4) |  | Prop haz model p=0.008  p<0.05 Z to P  p<0.05 Tema to P |
|  |  | Sleep latency in minutes Zolpi n=77 Tria n=75 Tema n=76 placebo n=75 at week 4 |  | 40.5 (3.1) vs 47.7 (3.5) vs 38.0 (3.0) vs 57.9 (5.6) |  | Prop haz model p=0.00  p<0.05 Z to P  p<0.05 Tema to P |
|  |  | Sleep latency in minutes Zolpi n=77 Tria n=75 Tema n=76 placebo n=75 at week 4 mean difference |  | -39.7 (4.7) vs  -39.4 (5.0) vs  -39.4 (4.7) vs  -21.4 (4.6) |  | Prop haz model p=0.011  p<0.05 Z to P  p<0.05 Tema to P |
|  |  | TST in minutes Zolpi n=82 Tria n=85 Tema n=84 placebo n=84 at baseline |  | 294.5 (6.9) vs 291.9 (6.4) vs 312.4 (5.4) vs 309.2 (5.8) |  | Prop haz model p=0.030 |
|  |  | TST in minutes Zolpi n=82 Tria n=85 Tema n=83 placebo n=83 at week 1 |  | 353.4 (6.1) vs 351.4 (7.1) vs 375.0 (7.0) vs 347.4 (7.2) |  | Prop haz model p=0.021  p<0.001 Tema to base |
|  |  | TST in minutes Zolpi n=82 Tria n=85 Tema n=83 placebo n=83 at week 1 mean difference |  | 58.9 (5.6) vs 59.5 (5.6) vs 62.7 (6.3) vs 38.1 (8.5) |  | Prop haz model p=0.023  p<0.05 Z to P  p<0.05 Tria to P  p<0.05 Tema to P |
|  |  | TST in minutes Zolpi n=79 Tria n=79 Tema n=77 placebo n=79 at week 2 |  | 356.2 (6.8) vs 358.6 (7.0) vs 364.1 (7.7) vs 354.8 (8.0) |  | Prop haz model p=0.772 |
|  |  | TST in minutes Zolpi n=79 Tria n=79 Tema n=77 placebo n=79 at week 2 mean difference |  | 60.7 (6.6) vs 70.3 (6.1) vs 49.7 (8.6) vs 45.3 (6.9) |  | Prop haz model p=0.02  p<0.05 Tria to P |
|  |  | TST in minutes Zolpi n=78 Tria n=78 Tema n=77 placebo n=78 at week 3 |  | 357.5 (7.0) vs 365.0 (6.8) vs 380.2 (7.5) vs 351.2 (8.8) |  | Prop haz model p=0.070 |
|  |  | TST in minutes Zolpi n=78 Tria n=78 Tema n=77 placebo n=78 at week 3 mean difference |  | 53.2 (7.5) vs 77.0 (6.4) vs 65.8 (8.1) vs 43.5 (7.7) |  | Prop haz model p=0.014  p<0.05 Tria to P |
|  |  | TST in minutes Zolpi n=77 Tria n=75 Tema n=76 placebo n=75 at week 4 |  | 362.8 (7.4) vs 359.7 (7.4) vs 375.3 (6.7) vs 363.0 (8.9) |  | Prop haz model p=0.573 |
|  |  | TST in minutes Zolpi n=77 Tria n=75 Tema n=76 placebo n=75 at week 4 mean difference |  | 70.0 (7.4) vs 72.7 (6.7) vs 61.8 (6.4) vs 51.8 (8.0) |  | Prop haz model p=0.090 |
|  |  | Overall adverse events Zolpidem vs Triazolam vs Temazepam vs placebo |  |  | n (%)  52 (63.0) vs 54 (64.0) vs 56 (67.0) vs 47 (56.0) |  |
|  |  | Headache Zolpidem vs Triazolam vs Temazepam vs placebo |  |  | n (%)  15 (18.3) vs 22 (25.9) vs 18 (21.4) vs 16 (19.0) |  |
|  |  | Myalgia Zolpidem vs Triazolam vs Temazepam vs placebo |  |  | n (%)  8 (9.8) vs 7 (8.2) vs 8 (9.5) vs 9 (10.7) |  |
|  |  | Drowsiness Zolpidem vs Triazolam vs Temazepam vs placebo |  |  | n (%)  4 (4.9) vs 7 (8.2) vs 10 (11.9) vs 3 (3.6) |  |
|  |  | Nausea Zolpidem vs Triazolam vs Temazepam vs placebo |  |  | n (%)  6 (7.3) vs 6 (7.1) vs 4 (4.8) vs 6 (7.1) |  |
|  |  | Upper resp infection Zolpidem vs Triazolam vs Temazepam vs placebo |  |  | n (%)  6 (7.3) vs 2 (2.4) vs 7 (8.3) vs 7 (8.3) |  |
|  |  | Dyspepsia Zolpidem vs Triazolam vs Temazepam vs placebo |  |  | n (%)  5 (6.1) vs 3 (3.5) vs 5 (6.0) vs 7 (8.3) |  |
|  |  | Nervousness Zolpidem vs Triazolam vs Temazepam vs placebo |  |  | n (%)  2 (2.4) vs 7 (8.2) vs 3 (3.6) vs 4 (4.8) |  |
|  |  | Arthralgia Zolpidem vs Triazolam vs Temazepam vs placebo |  |  | n (%)  4 (4.9) vs 5 (5.9) vs 0 vs 3 (3.6) |  |
|  |  | Fatigue Zolpidem vs Triazolam vs Temazepam vs placebo |  |  | n (%)  1 (1.2) vs 2 (2.4) vs 5 (6.0) vs 1 (1.2) |  |
| Roger 1993 (72) | Zolpidem 5/10 mg, Triazolam 0.25 mg | Drop-out due to lack of effect Zolpidem 5mg vs Zolpidem 10mg vs Triazolam |  |  | n  5 vs 1 vs 1 |  |
|  |  | Drop-out due to adverse effects (nightmares with sensation of imminent death, malaise with generalized tremor) Zolpidem 5mg vs Zolpidem 10mg vs Triazolam |  |  | n  0 vs 0 vs 2 |  |
|  |  | Easy sleep onset day 3 Zolpidem 5mg vs Zolpidem 10mg vs Triazolam |  |  | 16.2% vs 23.3% vs 20.8% |  |
|  |  | Easy sleep onset day 24 Zolpidem 5mg vs Zolpidem 10mg vs Triazolam |  |  | 72.1% vs 71.2% vs 72.7% |  |
|  |  | Easy sleep onset day 31 Zolpidem 5mg vs Zolpidem 10mg vs Triazolam |  |  | 50.8% vs 43.1% vs 39.4% |  |
|  |  | TST in h day 3 Zolpidem 5mg vs Zolpidem 10mg vs Triazolam |  |  | 5.2 vs 5.2 vs 5.3 |  |
|  |  | TST in h day 24 Zolpidem 5mg vs Zolpidem 10mg vs Triazolam |  |  | 6.8 vs 7.1 vs 7.2 |  |
|  |  | >2 nocturnal awakenings day 3 Zolpidem 5mg vs Zolpidem 10mg vs Triazolam |  |  | 61.8% vs 58.9% vs 59.7% |  |
|  |  | >2 nocturnal awakenings day 24 Zolpidem 5mg vs Zolpidem 10mg vs Triazolam |  |  | 25.0% vs 30.1% vs 29.9% |  |
|  |  | >2 nocturnal awakenings day 31 Zolpidem 5mg vs Zolpidem 10mg vs Triazolam |  |  | 36.1% vs 48.6% vs 43.7% |  |
|  |  | >60 min nocturnal awakenings day 3 Zolpidem 5mg vs Zolpidem 10mg vs Triazolam |  |  | 55.9% vs 47.9% vs 55.8% |  |
|  |  | >60 min nocturnal awakenings day 24 Zolpidem 5mg vs Zolpidem 10mg vs Triazolam |  |  | 17.6% vs 11.0% vs 15.6% |  |
|  |  | >60 min nocturnal awakenings day 31 Zolpidem 5mg vs Zolpidem 10mg vs Triazolam |  |  | 13.6% vs 29.6% vs 26.4% |  |
|  |  | Quality of sleep 0=very poor, 100=very well day 3 Zolpidem 5mg vs Zolpidem 10mg vs Triazolam |  | Mean (SD)  27.6 (2.8) vs 29.4 (3.0) vs 27. 8(2.7) |  |  |
|  |  | Quality of sleep 0=very poor, 100=very well day 24 Zolpidem 5mg vs Zolpidem 10mg vs Triazolam |  | Mean (SD)  63.1 (3.3) vs 62.2 (3.2) vs 61.4 (3.3) |  |  |
|  |  | Rescue hypnotic at least once Zolpidem 5mg vs Zolpidem 10mg vs Triazolam |  |  | n  11 vs 10 vs 7 |  |
|  |  | Clinical global impression scale Zolpidem 5mg vs Zolpidem 10mg vs Triazolam |  | Mean (SD n.r.)  2.54 vs 2.43 vs 2.51 |  |  |
|  |  | Overall adverse events Zolpidem 5mg vs Zolpidem 10mg vs Triazolam |  |  | n  11 vs 8 vs 16 |  |
|  |  | Nightmares Zolpidem 5mg vs Zolpidem 10mg vs Triazolam |  |  | n  2 vs 3 vs 2 |  |
|  |  | Agitation Zolpidem 5mg vs Zolpidem 10mg vs Triazolam |  |  | n  1 vs 3 vs 2 |  |
|  |  | Falls Zolpidem 5mg vs Zolpidem 10mg vs Triazolam |  |  | n  0 vs 0 vs 1 |  |
|  |  | Drowsiness or impaired concentration Zolpidem 5mg vs Zolpidem 10mg vs Triazolam |  |  | n  2 vs 0 vs 1 |  |
|  |  | Weakness of legs Zolpidem 5mg vs Zolpidem 10mg vs Triazolam |  |  | n  0 vs 0 vs 1 |  |
|  |  | Visual disturbance Zolpidem 5mg vs Zolpidem 10mg vs Triazolam |  |  | n  1 vs 0 vs 0 |  |
| Scharf 2005 (73) | Eszopiclone 1/2 mg, placebo | Sleep latency, min experimental phase Eszopiclone 2mg vs placebo |  | Mean (SD)  50.0 (56) vs 85.5 (97) |  | p=0.0034 |
|  |  | TST, min experimental phase Eszopiclone 2mg vs placebo |  | Mean (SD)  372.3 (72) vs 328.2 (88) |  | p=0.0003 |
|  |  | WASO, min experimental phase Eszopiclone 2mg vs placebo |  | Mean (SD) 58.5 (50) vs 74.1 (56) |  | p=0.0423 |
|  |  | Awakenings, n/night experimental phase Eszopiclone 2mg vs placebo |  | Mean (SD)  1.7 (1.0) vs 1.9 (0.9) |  | p<0.05 |
|  |  | Sleep quality 11-point Likert scale, experimental phase Eszopiclone 2mg vs placebo |  | Mean (SD)  7.2 (1.6) vs 6.3 (1.8) |  | p=0.0006 |
|  |  | Sleep depth 11-point Likert scale, experimental phase Eszopiclone 2mg vs placebo |  | Mean (SD)  7.1 (1.6) vs 6.2 (1.8) |  | p=0.0015 |
|  |  | Sleep latency, min change from baseline experimental phase Eszopiclone 2mg vs placebo |  | Mean (SD)  -70.0 (175) vs  -41.4 (146) |  | p=0.0059 |
|  |  | TST, min change from baseline experimental phase Eszopiclone 2mg vs placebo |  | Mean (SD) 87.0 (142) vs 38.1 (124) |  | p=0.0002 |
|  |  | WASO, min change from baseline experimental phase Eszopiclone 2mg vs placebo |  | Mean (SD)  -39.9 (79) vs  8.3 (65) |  | p=0.0009 |
|  |  | Awakenings , n/night change from baseline experimental phase Eszopiclone 2mg vs placebo |  | Mean (SD)  -0.8 (1.3) vs  -0.1 (1.2) |  | p=0.0170 |
|  |  | Sleep quality 11-point Likert scale, change from baseline experimental phase Eszopiclone 2mg vs placebo |  | Mean (SD)  2.2 (2.4) vs 0.9 (2.7) |  | p=0.0018 |
|  |  | Sleep depth 11-point Likert scale, change from baseline experimental phase Eszopiclone 2mg vs placebo |  | Mean (SD)  2.5 (2.4) vs 1.2 (2.4) |  | p=0.0064 |
|  |  | Daytime alertness 11-point Likert scale experimental phase Eszopiclone 2mg vs placebo |  | Mean (SD)  7.3 (1.6) vs 6.8 (1.6) |  | p=0.0223 |
|  |  | Physical well being 11-point Likert scale experimental phase Eszopiclone 2mg vs placebo |  | Mean (SD)  7.7 (1.6) vs 7.2 (1.6) |  | p=0.0474 |
|  |  | Morning sleepiness 11-point Likert scale experimental phase Eszopiclone 2mg vs placebo |  | Mean (SD)  7.2 (1.7) vs 6.6 (1.9) |  | p=0.0547 |
|  |  | Ability to function 11-point Likert scale experimental phase Eszopiclone 2mg vs placebo |  | Mean (SD)  7.6 (1.7) vs 7.2 (1.6) |  | p=0.0579 |
|  |  | Naps taken total n experimental phase Eszopiclone 2mg vs placebo |  | Mean (SD)  4.3 (3.4) vs 5.9 (4.0) |  | p=0.0276 |
|  |  | Duration per nap, experimental phase Eszopiclone 2mg vs placebo |  | Mean (SD)  2.7 (3.7) vs 59.2 (51) |  | p=0.0113 |
|  |  | Q-LES-Q Eszo 2mg vs placebo higher global score |  |  |  | p=0.0639 |
|  |  | Total adverse events Eszo 2mg vs placebo |  |  | 43% vs 40% |  |
|  |  | Discontinuation due to adverse events Eszo 2mg vs placebo |  |  | 2.5% vs 6.3% |  |
|  |  | Headache Eszo 2mg vs placebo |  |  | 15.2% vs 15.0% |  |
|  |  | Unpleasant taste Eszo 2mg vs placebo |  |  | 11.4% vs 1.3% |  |
|  |  | Somnolence Eszo 2mg vs placebo |  |  | 3.8% vs 8.8% |  |
|  |  | Dyspepsia Eszo 2mg vs placebo |  |  | 1.3% vs 2.5% |  |
