# Supplementary material for: Efficacy and safety of Z-substances in the management of insomnia in older adults: a systematic review for the development of recommendations to reduce potentially inappropriate prescribing
Source: BMC Geriatr. 2022 Feb 1;22:87. doi: 10.1186/s12877-022-02757-6 (PMC9887772; doi:10.1186/s12877-022-02757-6)
Supplement: Supplementary file 5 — Additional file 5. Findings Observational Studies [file 12877_2022_2757_MOESM5_ESM.docx]

**Additional file 5: Findings: observational studies**

| Reference | Study medication/ comparator | Outcomes | RR, OR, HR | P |
| --- | --- | --- | --- | --- |
| Avidan 2010 (83) | BDLM  (Zolpidem, Zaleplon, Eszopiclone)  Selective melatonin receptor agonist (Ramelteon) | Risk of accidental event compared to melatonin receptor agonist cohort 1-month follow-up BDLM treatment vs Melatonin receptor agonist | aOR 1.12 (CI95 n.r.)  [184 of 156987 vs 0 of 988, crude OR 1.16 (CI950.16-8.27)] | p<0.05 |
|  |  | Risk of accidental event compared to melatonin receptor agonist cohort 3-month follow-up BDLM treatment vs Melatonin receptor agonist | aOR 1.48 (CI95 n.r.)  [440 of 156987 vs 0 of 988, crude OR 2.77 (CI95 0.38-19.76)] | p<0.05 |
| Berry 2013 (74) | Dispensing of BDLM (Zolpidem, Eszopiclone, Zaleplon) | Risk for hip fracture any BDLM hypnotic drug use 0-15 d before hip fracture n=622 | OR (CI95)  1.47 (1.24-1.74) |  |
|  |  | Risk for hip fracture any BDLM hypnotic drug use 0-30 d before hip fracture n=927 | OR (CI95)  1.66 (1.45-1.90) |  |
|  |  | Risk for hip fracture new BDLM hypnotic drug use 0-15 d before hip fracture n=366 | OR (CI95)  2.20 (1.76-2.74) |  |
|  |  | Risk for hip fracture new BDLM hypnotic drug use 0-30 d before hip fracture n=564 | OR (CI95)  1.90 (1.60-2.26) |  |
| Chang 2011 (75) | Zolpidem falls vs no falls | Risk for falls in in-hospital setting with Zolpidem use 24 hours before fall | OR (CI95)  2.38 (1.04–5.43) | p=0.040 |
| Kang 2012 (76) | Prescription of Zolpidem | Risk of fracture from Zolpidem use, Zolpidem hazard exposure n=236 of 1508 vs control period exposures n= 722 of 6032 (logistic regression) | OR (CI95)  1.84 (1.47-2.30) |  |
|  |  | Risk of fracture from Zolpidem use, Zolpidem hazard exposure n=236 control period exposures n= 722, adjusted for analgesics, anticholinergics, antipsychotics, antiepileptics, and calcium channel blockers | OR (CI95)  1.72 (1.37-2.16) |  |
|  |  | Risk of fracture from Zolpidem exposure, aged 65-69 n =302 | OR (CI95)  1.90 (1.17-3.11) |  |
|  |  | Risk of fracture from Zolpidem exposure, aged 70-74 n=407 | OR (CI95)  1.69 (1.10-2.59) |  |
|  |  | Risk of fracture from Zolpidem exposure, aged 75-79 n=411 | OR (CI95)  1.56 (1.00-2.45) |  |
|  |  | Risk of fracture from Zolpidem exposure, aged 80-84 n=253 | OR (CI95)  1.69 (1.00-2.86) |  |
|  |  | Risk of fracture from Zolpidem exposure, aged 85 and over n=135 | OR (CI95)  4.48 (2.00-10.04) |  |
|  |  | Risk of fracture from Zolpidem exposure, male n=302 | OR (CI95)  1.74 (1.09-2.77) |  |
|  |  | Risk of fracture from Zolpidem exposure, female n=1206 | OR (CI95)  1.87 (1.45-2.41) |  |
| Lai 2015 (77) | Zopiclone exposure prior to fracture vs no exposure prior to fracture | Risk of hip fracture from current Zopiclone user | OR (CI95)  3.87 (2.71–5.53) |  |
|  |  | Risk of hip fracture from current Zopiclone user adjusted for age, benzodiazepine use, number of medications, cancer, cardiovascular disease, chronic kidney disease, dementia, depression, and Parkinson’s disease | OR (CI95)  3.56 (2.33–4.84) |  |
|  |  | Risk of hip fracture from late Zopiclone user | OR (CI95)  1.12 (1.00–1.25) |  |
|  |  | Risk of hip fracture from late Zopiclone user adjusted for age, benzodiazepine use, number of medications, cancer, cardiovascular disease, chronic kidney disease, dementia, depression, and Parkinson’s disease | OR (CI95)  1.05 (0.94–1.18) |  |
| Pierfitte 2001 (78) | Zopiclone or Zolpidem exposure | Risk of hip fracture in Zolpidem users vs non-users | OR (CI95)  1.3 (0.7-2.5)  [15/245 vs 35/817, crude OR 1.46  (CI95 0.78-2.72)] |  |
|  |  | Risk of hip fracture in Zopiclone users vs non-users | OR (CI95)  0.7 (0.4-1.4) |  |
| Tang 2015 (79) | Prescription of Zolpidem | Risk of fracture due to use of Zolpidem cases n= 487 of 6010 vs control cases n=1755 of 24040 (logistic regression) | OR (CI95)  1.27 (1.09-1.48) | p<0.05 |
|  |  | Risk of fracture due to use of Zolpidem cases n= 487 control cases n=1755 adjusted for use of antidepressants, antipsychotics and diuretics | OR (CI95)  1.23 (1.06-1.44) | p<0.05 |
|  |  | Risk of fracture due to use of Zolpidem cases n= 487 control cases n=1755 adjusted for hypertension, osteoarthritis, osteoporosis, rheumatoid arthritis and depression | OR (CI95)  1.13 (0.96-1.34) | p=0.136 |
|  |  | Risk of fracture due to use of Zolpidem age 65-74 n=788 | OR (CI95)  1.27 (1.08-1.48) | p<0.05 |
|  |  | Risk of fracture due to use of Zolpidem age 65-74 n=788 adjusted for hypertension, osteoarthritis, osteoporosis, rheumatoid arthritis and depression | OR (CI95)  1.16 (0.89-1.52) | p=0.274 |
|  |  | Risk of fracture due to use of Zolpidem age 75-84 n=1177 | OR (CI95)  1.44 (1.20-1.73) | p<0.05 |
|  |  | Risk of fracture due to use of Zolpidem age 75-84 n=1177 adjusted for hypertension, osteoarthritis, osteoporosis, rheumatoid arthritis and depression | OR (CI95)  1.16 (0.92-1.45) | p=0.227 |
|  |  | Risk of fracture due to use of Zolpidem age 85 and older n=277 | OR (CI95)  1.32 (0.91-1.93) | p=0.148 |
|  |  | Risk of fracture due to use of Zolpidem age 85 and older n=277 adjusted for hypertension, osteoarthritis, osteoporosis, rheumatoid arthritis and depression | OR (CI95)  0.98 (0.61-1.58) | p=0.919 |
|  |  | Risk of fracture due to use of Zolpidem male n=754 | OR (CI95)  1.41 (1.15-1.73) | p<0.05 |
|  |  | Risk of fracture due to use of Zolpidem male n=754 adjusted for hypertension, osteoarthritis, osteoporosis, rheumatoid arthritis and depression | OR (CI95)  1.05 (0.79-1.39) | p=0.793 |
|  |  | Risk of fracture due to use of Zolpidem female n=1488 | OR (CI95)  1.31 (1.14-1.50) | p<0.001 |
|  |  | Risk of fracture due to use of Zolpidem female n=1488 adjusted for hypertension, osteoarthritis, osteoporosis, rheumatoid arthritis and depression | OR (CI95)  1.18 (0.96-1.45) | p=0.108 |
|  |  | Risk of fracture due to use of Zolpidem hypertension yes n=1599 | OR (CI95)  1.26 (1.05-1.51) | p<0.05 |
|  |  | Risk of fracture due to use of Zolpidem hypertension yes n=1599 adjusted for hypertension, osteoarthritis, osteoporosis, rheumatoid arthritis and depression except for the variable of own strata | OR (CI95)  1.17 (0.96-1.41) | p=0.121 |
|  |  | Risk of fracture due to use of Zolpidem hypertension no n=643 | OR (CI95)  1.17 (0.87-1.56) | p=0.305 |
|  |  | Risk of fracture due to use of Zolpidem hypertension no n=643 adjusted for hypertension, osteoarthritis, osteoporosis, rheumatoid arthritis and depression except for the variable of own strata | OR (CI95)  1.05 (0.76-1.45) | p=0.755 |
|  |  | Risk of fracture due to use of Zolpidem osteoarthritis yes n=1104 | OR (CI95)  1.13 (0.91-1.40) | p=0.270 |
|  |  | Risk of fracture due to use of Zolpidem osteoarthritis yes n=1104 adjusted for hypertension, osteoarthritis, osteoporosis, rheumatoid arthritis and depression except for the variable of own strata | OR (CI95)  1.06 (0.85-1.33) | p=0.603 |
|  |  | Risk of fracture due to use of Zolpidem osteoarthritis no n=1138 | OR (CI95)  1.36 (1.08-1.71) | p<0.05 |
|  |  | Risk of fracture due to use of Zolpidem osteoarthritis no n=1138 adjusted for hypertension, osteoarthritis, osteoporosis, rheumatoid arthritis and depression except for the variable of own strata | OR (CI95)  1.23 (0.96-1.57) | p=0.101 |
|  |  | Risk of fracture due to use of Zolpidem osteoporosis yes n=1926 | OR (CI95)  0.95 (0.62-1.45) | p=0.817 |
|  |  | Risk of fracture due to use of Zolpidem osteoporosis yes n=1926 adjusted for hypertension, osteoarthritis, osteoporosis, rheumatoid arthritis and depression except for the variable of own strata | OR (CI95)  0.96 (0.62-1.48) | p=0.842 |
|  |  | Risk of fracture due to use of Zolpidem osteoporosis no n=316 | OR (CI95)  1.28 (1.08-1.52) | p<0.05 |
|  |  | Risk of fracture due to use of Zolpidem osteoporosis no n=316 adjusted for hypertension, osteoarthritis, osteoporosis, rheumatoid arthritis and depression except for the variable of own strata | OR (CI95)  1.17 (0.98-1.40) | p=0.089 |
|  |  | Risk of fracture due to use of Zolpidem Rheumatid arthritis yes n=62 | OR (CI95)  0.89 (0.36-2.18) | p=0.792 |
|  |  | Risk of fracture due to use of Zolpidem Rheumatid arthritis yes n=62 adjusted for hypertension, osteoarthritis, osteoporosis, rheumatoid arthritis and depression except for the variable of own strata | OR (CI95)  0.76 (0.29-1.99) | p=0.571 |
|  |  | Risk of fracture due to use of Zolpidem Rheumatid arthritis no n=2180 | OR (CI95)  1.28 (1.10-1.49) | p<0.05 |
|  |  | Risk of fracture due to use of Zolpidem Rheumatid arthritis no n=2180 adjusted for hypertension, osteoarthritis, osteoporosis, rheumatoid arthritis and depression except for the variable of own strata | OR (CI95)  1.15 (0.97-1.36) | p=0.106 |
|  |  | Risk of fracture due to use of Zolpidem depression yes n=533 | OR (CI95)  0.86 (0.60-1.23) | p=0.397 |
|  |  | Risk of fracture due to use of Zolpidem depression yes n=533 adjusted for hypertension, osteoarthritis, osteoporosis, rheumatoid arthritis and depression except for the variable of own strata | OR (CI95)  0.87 (0.59-1.28) | p=0.478 |
|  |  | Risk of fracture due to use of Zolpidem depression no n=1709 | OR (CI95)  1.36 (1.14-1.61) | p<0.001 |
|  |  | Risk of fracture due to use of Zolpidem depression no n=1709 adjusted for hypertension, osteoarthritis, osteoporosis, rheumatoid arthritis and depression except for the variable of own strata | OR (CI95)  1.21 (1.00-1.45) | p<0.05 |
|  |  | Risk of hip fracture due to use of Zolpidem | OR (CI95)  1.53 (1.04-2.25) | p<0.05 |
|  |  | Risk of hip fracture due to use of Zolpidem adjusted for use of antidepressants, antipsychotics and diuretics | OR (CI95)  1.49 (1.00-2.22) | p<0.05 |
|  |  | Risk of hip fracture due to use of Zolpidem adjusted for hypertension, osteoarthritis, osteoporosis, rheumatoid arthritis and depression | OR (CI95)  1.25 (0.83-1.87) |  |
|  |  | Risk of humerus fracture due to use of Zolpidem | OR (CI95)  1.28 (0.67-2.44) |  |
|  |  | Risk of humerus fracture due to use of Zolpidem adjusted for use of antidepressants, antipsychotics and diuretics | OR (CI95)  1.18 (0.61-2.29) |  |
|  |  | Risk of humerus fracture due to use of Zolpidem adjusted for hypertension, osteoarthritis, osteoporosis, rheumatoid arthritis and depression | OR (CI95)  1.20 (0.59-2.44) |  |
|  |  | Risk of forearm fracture due to use of Zolpidem | OR (CI95)  1.09 (0.70-1.70) |  |
|  |  | Risk of forearm fracture due to use of Zolpidem adjusted for use of antidepressants, antipsychotics and diuretics | OR (CI95)  1.08 (0.69-1.70) |  |
|  |  | Risk of forearm fracture due to use of Zolpidem adjusted for hypertension, osteoarthritis, osteoporosis, rheumatoid arthritis and depression | OR (CI95)  1.05 (0.66-1.69) |  |
|  |  | Risk of wrist fracture due to use of Zolpidem | OR (CI95)  2.00 (0.18-22.05) |  |
|  |  | Risk of wrist fracture due to use of Zolpidem adjusted for use of antidepressants, antipsychotics and diuretics | OR (CI95)  2.00 (0.18-22.05) |  |
|  |  | Risk of wrist fracture due to use of Zolpidem adjusted for hypertension, osteoarthritis, osteoporosis, rheumatoid arthritis and depression | OR (CI95)  4.00 (0.25-63.92) |  |
|  |  | Risk of spine fracture due to use of Zolpidem | OR (CI95)  1.38 (1.07-1.78) | p<0.05 |
|  |  | Risk of spine fracture due to use of Zolpidem adjusted for use of antidepressants, antipsychotics and diuretics | OR (CI95)  1.36 (1.05-1.76) | p<0.05 |
|  |  | Risk of spine fracture due to use of Zolpidem adjusted for hypertension, osteoarthritis, osteoporosis, rheumatoid arthritis and depression | OR (CI95)  1.23 (0.85-1.49) |  |
|  |  | Risk of other fracture due to use of Zolpidem | OR (CI95)  1.13 (0.87-2.46) |  |
|  |  | Risk of other fracture due to use of Zolpidem adjusted for use of antidepressants, antipsychotics and diuretics | OR (CI95)  1.25 (1.07-1.45) | p<0.05 |
|  |  | Risk of other fracture due to use of Zolpidem adjusted for hypertension, osteoarthritis, osteoporosis, rheumatoid arthritis and depression | OR (CI95)  1.13 (0.96-1.34) |  |
| Tom 2016 (80) | Prescription of Zaleplon, Zolpidem or Eszopiclone | Odds of TBI hospitalization associated with  BDLM use in past 30 days: Zolpidem | OR (CI95%)  1.87 (1.56-2.25) |  |
|  |  | Odds of TBI hospitalization associated with  BDLM use in past 30 days: Eszopiclone | OR (CI95%)  0.67 (0.40-1.13) |  |
|  |  | Odds of TBI hospitalization associated with  BDLM use in past 30 days: Zaleplon | OR (CI95%)  0.85 (0.21-3.34) |  |
|  |  | Odds of hip fracture hospitalization associated with BDLM use in past 30 days: Zolpidem | OR (CI95%)  1.59 (1.41-1.79) |  |
|  |  | Odds of hip fracture hospitalization associated with BDLM use in past 30 days: Eszopiclone | OR (CI95%)  1.12 (0.83-1.50) |  |
|  |  | Odds of hip fracture hospitalization associated with BDLM use in past 30 days: Zaleplon | OR (CI95%)  0.92 (0.40-2.13) |  |
| Wang 2001 (81) | Prescriptions for sedative hypnotics filled in the 6 months before the subjects’ index date | Risks of hospitalization for surgical repair of hip fracture Zolpidem vs no Zolpidem | aOR (CI95)  2.26 (1.28-3.97)  [20/1222 vs 34/4888, crude OR 2.38 (CI95 1.36-4.14)] |  |
|  |  | Risks of hospitalization for surgical repair of hip fracture Zolpidem vs no Zolpidem adjusted for age and gender | aOR (CI95)  1.95 (1.09-3.51) |  |
| Zint 2010 (82) | Use of Zolpidem in 14 days prior to hip fracture | Risk of hospitalization for hip fracture of patients using Zolpidem vs patients not using any BDZ or BDLM | aRR (CI95)  1.48 (1.32-1.66)  [456/2840 vs 1608/11410, crude OR 1.17 (CI95 1.04-1.31)] |  |
|  |  | Risk of hospitalization for hip fracture of patients using Zolpidem vs patients not using any BDZ or BDLM adjusted for age gender and race | aRR (CI95)  1.43 (1.26- 1.62) |  |
|  |  | Risk of hospitalization for hip fracture of patients using Zolpidem vs patients not using any BDZ or BDLM adjusted for age, gender, race, income, healthcare utilization, comorbidity, and comedication | aRR (CI95)  1.26 (1.11-1.44) |  |
